# Minimally Invasive and in Situ Capacitive Sensing of Cardiac Troponin I from Interstitial Fluid

**DOI:** 10.1021/acssensors.5c01691

**Published:** 2025-07-28

**Authors:** Hadi Mirzajani, Parviz Zolfaghari, Beril Yagmur Koca, Hakan Urey

**Affiliations:** † Department of Electrical and Electronics Engineering, 52979 Koç University, 34450 Istanbul, Türkiye; ‡ Koç University Research Center for Translational Medicine (KUTTAM), 34450 Istanbul, Türkiye

**Keywords:** biosensor, microneedle, capacitive sensing, wearable biosensing, cardiac biomarker, heart attack, cardiac troponin I

## Abstract

Current diagnostic approaches for myocardial infarction (MI) rely on blood-based cardiac biomarker analysis by centralized instruments, often delaying timely clinical decisions. We present a microneedle-based capacitive biosensor (MiCaP) for in situ minimally invasive monitoring of cardiac troponin I (cTnI) in interstitial fluid (ISF) for point-of-care (POC) applications. MiCaP is a label-free biosensor operating based on nonfaradaic sensing by monitoring electric double layer capacitance at the microneedle-ISF interface. We extracted a simplified equivalent circuit model for MiCaP inserted into the skin, confirming that the measured capacitance variations originate from cTnI binding to surface-immobilized antibodies. MiCaP was fabricated by using a scalable process and functionalized with anti-cTnI antibodies. In vitro measurements showed a dynamic detection range of 10 pg/mL to 10 ng/mL, a limit of detection (LOD) of 3.27 pg/mL, and a total assay response time of less than 15 min. A spike-and-recovery test using cTnI-spiked human serum yielded a recovery accuracy exceeding 93%. In vivo studies in rats demonstrated ISF cTnI levels of 3 ± 0.4 pg/mL in controls and 912 ± 683 pg/mL in experimental animals, indicating an increasing trend consistent with serum concentrations measured using a clinical immunoassay. These results support the potential of MiCaP as a minimally invasive biosensing platform for cardiac biomarker monitoring, with possible extension to multiplexed ISF-based diagnostics in the POC.

## Graphical abstract

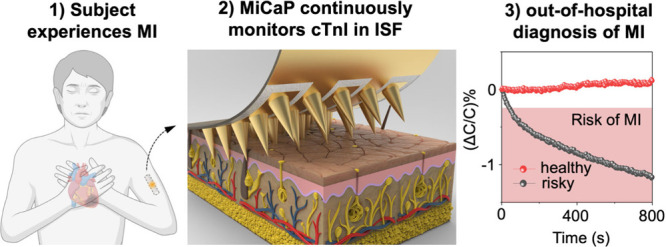

According to the World Health Organization (WHO), cardiovascular diseases (CVDs) account for approximately 17.9 million fatalities annually, constituting 32% of all deaths.^1,2^ Among CVDs, myocardial infarction (MI) presents a significant health challenge and incurs substantial economic costs on global healthcare systems, with annual expenses of around €200 billion in the EU and $108 billion in the US.^3,4^ Standard medical guidelines emphasize the importance of diagnosing the possibility of an MI within the early hours of symptom onset and seeking prompt medical attention.^2,5–7^ However, current medical practices require patient admission to the emergency department (ED), followed by invasive blood sampling and quantification of cardiac biomarker concentrations through labor-intensive and centralized laboratory-based techniques. These methods require trained personnel and involve prolonged response times.^2,6,8^ These shortcomings can lead to late MI diagnosis, leading to increased morbidity, heightened mortality, and reduced efficacy of interventional and pharmacologic treatments.^2^ Therefore, there is a need for a point-of-care (POC) diagnostic platform that enables cardiac biomarker monitoring outside hospital settings, at home, or in ambulatory setups.^9–13^

The cTnI is a key biomarker for the diagnosis of MI and is widely used in clinical practice, including hospitals and emergency departments.^14,15^ Previously demonstrated cTnI biosensors are mostly focused on the surface modification of electrodes to enhance sensitivity and lower the LOD, which can reach values as low as 28.1 ag/mL^16^ (Table S1). However, these methods depend on blood sampling, complex surface functionalization techniques, and the use of benchtop instrumentation for signal reading,^16–18^ which is not suitable for POC applications.

In recent years, microneedle-based minimally invasive wearable biosensing systems have facilitated patient-centered and remote health monitoring through in situ biomarker analysis, marking a paradigm shift in personalized medicine.^19–21^ These devices have attracted significant interest owing to the compositional similarity of interstitial fluid (ISF) to blood in terms of proteomic and metabolomic content and its minimally invasive accessibility.^22–25^ Initial microneedle-based biomarker detection focused on extracting ISF by microneedles (hollow or hydrogel-based microneedles), followed by ISF analysis over external biosensors.^22,23,26^ However, ISF extraction requires instruments to generate negative pressure (creating complexities in system integration), takes a long time (extraction of 5 μL ISF takes more than 1 h), and yields a lower concentration of the target biomarker in extracted ISF (the mesh-like structure of the dermis acts as a filter for large molecules).^23^ In situ molecule capture with ex vivo detection has been adopted to address these limitations, requiring laboratory-based instruments for off-body quantification, which may not be suitable for POC applications.^22,28^ On the other hand, in situ monitoring is gaining increasing attention for its potential in POC diagnostic systems.^21,29–33^ The in situ technique has already been successfully implemented for the detection of various biomarkers, including ions (Na^+^, K^+^, Ca^2+^, Li^+^),^29,34^ tyrosinase,^29,34^ dopamine,^35^ lactate,^21^ glucose,^21^ and alcohol,^21^ as well as more complex targets such as cell-free DNA and RNA targets,^37^ bovine serum albumin,^38^ and nitric oxide.^39^ However, the majority of microneedle biosensors for in situ monitoring rely on faradaic electrochemical detection mechanisms, which depend on enzymatic reactions, redox-active species, or labeled molecular probes, such as electrochemical aptamer (EAB) biosensors (a comprehensive list is provided in Table S2). While effective, these approaches introduce several challenges. Enzymatic components are often unstable, especially under ambient or physiological conditions,^40^ leading to a limited operational lifetime. Labeled probes require complex fabrication steps and may interfere with target accessibility.^41^ Additionally, redox-active detection schemes typically require external mediators and are more susceptible to signal drift and interference from endogenous electroactive species in interstitial fluid.^42^ In contrast, nonfaradaic detection strategies based on EDL capacitance interrogation offer a label-free, redox-free, and stable alternative to faradaic sensing. By monitoring interfacial capacitance changes induced by biomolecular interactions at the microneedle–ISF interface, where probe molecules are immobilized, these sensors eliminate the need for enzymatic amplification or redox mediators. This simplifies the device architecture, making them particularly well-suited for in situ measurements. Despite these advantages, few microneedle biosensors have implemented nonfaradaic capacitive sensing architectures.^43,44^ We previously reported a proof-of-concept nonfaradaic biosensor for BSA detection by microneedles.^38^

Here in this study, we present MiCaP, a microneedle-integrated capacitive biosensor designed for in situ monitoring of cTnI in ISF (Figure 1a). To the best of our knowledge, this is the first microneedle biosensor for in situ cTnI detection in ISF. The device incorporates interdigitated electrodes (IDE) with five fingers, each connected to five microneedles in a row (Figure 1b), enabling spatially distributed capacitive biosensing. The capacitive nature of MiCaP was verified by extracting and simplifying its equivalent circuit model under in-skin conditions. The microneedle surface was functionalized with a cTnI-specific antibody (Figure 1c,d), and the normalized percentage change in capacitance (%Δ*C*/*C*) was used as the analytical output to improve the response consistency (Figure 1e). Analytical characterization demonstrated a dynamic range spanning 4 orders of magnitude (10 pg/mL to 10 ng/mL), a low LOD of 3.27 pg/mL, and a total assay response time under 15 min. Quantitative accuracy was validated via a spike-and-recovery assay in cTnI-free human serum, achieving recovery rates exceeding 93%. In vivo experiments in a rat model further demonstrated MiCaP’s ability to track physiologically relevant cTnI levels. As summarized in Table S1, MiCaP demonstrates comparable performance with respect to previously reported cTnI biosensing platforms in terms of LOD and response time. While several biosensing approaches have reported lower LOD and faster response times, the MiCaP platform enables in situ monitoring of cTnI directly from ISF through a minimally invasive microneedle interface. Its core advantage lies in its compatibility with continuous and in vivo sensing applications. Overall, this work advances the development of wearable, minimally invasive biosensors and contributes to the global health objectives outlined in the United Nations Sustainable Development Goal 3.4, which emphasizes early detection and management of noncommunicable diseases.

**Figure 1:**
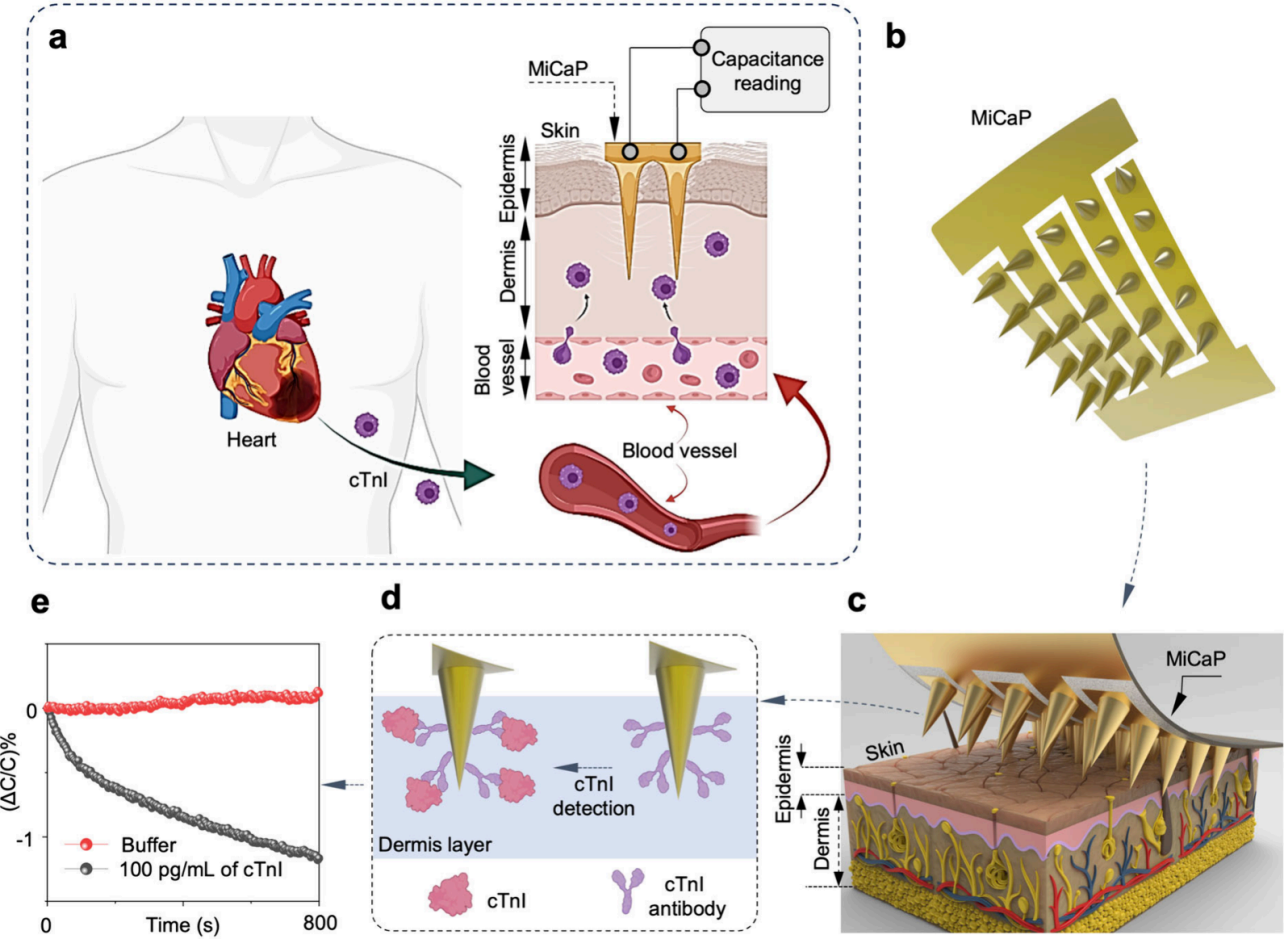
Schematic overview of the MiCaP for the in situ detection and quantification of dermal cTnI. (a) cTnI, released from the heart during MI events, transitions from the bloodstream into the ISF through capillary endothelial cells. Upon insertion of the functionalized MiCaP into the dermal layer, microneedles capture cTnI from ISF, and the concentration is quantified via capacitance interrogation of the MiCaP using a capacitance reading circuit. (b) Schematic illustration of the MiCaP design, showing the microneedle array and the interdigitated electrode pattern. (c) Three-dimensional illustration of MiCaP inserted into skin tissue. (d) Schematic of a single microneedle modified with antibodies for specific cTnI detection in dermis. (e) Representative variation of the sensor’s output metric (Δ*C*/*C*%), demonstrating MiCaP’s response to cTnI.

## Results and Discussion

### Structure and Initial Characterizations

The device includes a 5 × 5 array of conical microneedles fabricated from poly­(lactic acid) (PLA) through replica molding. This array was used to ensure a sufficient electrochemical surface area for robust and repeatable capacitance measurements. This configuration enhances signal stability by averaging responses across multiple microneedles. Each microneedle has a base diameter of 600 μm, a height of 1.5 mm, and a 600 μm spacing between adjacent microneedles (Figure 2a and b). A Cr/Au (50/200 nm) layer was deposited by the shadow masking technique to establish an interdigitated configuration over microneedles, facilitating capacitive biosensing (Figure 2c). A detailed fabrication process flow is provided in Figure S1, and Video S1 shows the final fabricated microneedle array. The flexibility of the fabricated device is shown in Figure 2d. To examine the proper Cr/Au deposition and electrical connection, a probe station was employed to evaluate the electrical resistance of single microneedles from the tip to the pad (Figure 2e), revealing consistent resistance values below 100 Ω across all microneedles. Following fabrication, the base of the microneedles was coated with a thin layer of polydimethylsiloxane (PDMS) to ensure that only the tips (∼500 μm) of the microneedles are exposed to ISF (Figure 2f). The electrochemical performance and reproducibility of MiCaP were evaluated using cyclic voltammetry (CV) and electrochemical impedance spectroscopy (EIS) by an Autolab PGSTAT 101 instrument across three independently fabricated and cleaned sensors. CV measurements were performed in 5 mM K_3_[Fe­(CN)_6_] prepared in PBS (pH 7.4) using external reference and counter electrodes. The recorded voltammograms exhibited consistent redox behavior, with anodic peak currents averaging approximately 1.5 mA across all samples, indicating repeatable electrochemical performance (Figure 2g). EIS measurements were conducted by a LCX meter (R&SLCX200, Rohde and Schwarz) in 0.1 × PBS to assess baseline capacitive behavior. Minimal variation was observed among the three sensors, supporting the reproducibility of the fabrication process (Figure 2h). In addition, continuous capacitance monitoring of MiCaP exposed to 0.1 × PBS was performed using the impedance analyzer over a duration of 1800 s. The recorded capacitance showed stable behavior, with a mean value of 50.14 × 10^–9^ F and a standard deviation of 11.42 × 10^–11^ F, further confirming the stability and reproducibility of the sensor (Figure 2i).

**Figure 2:**
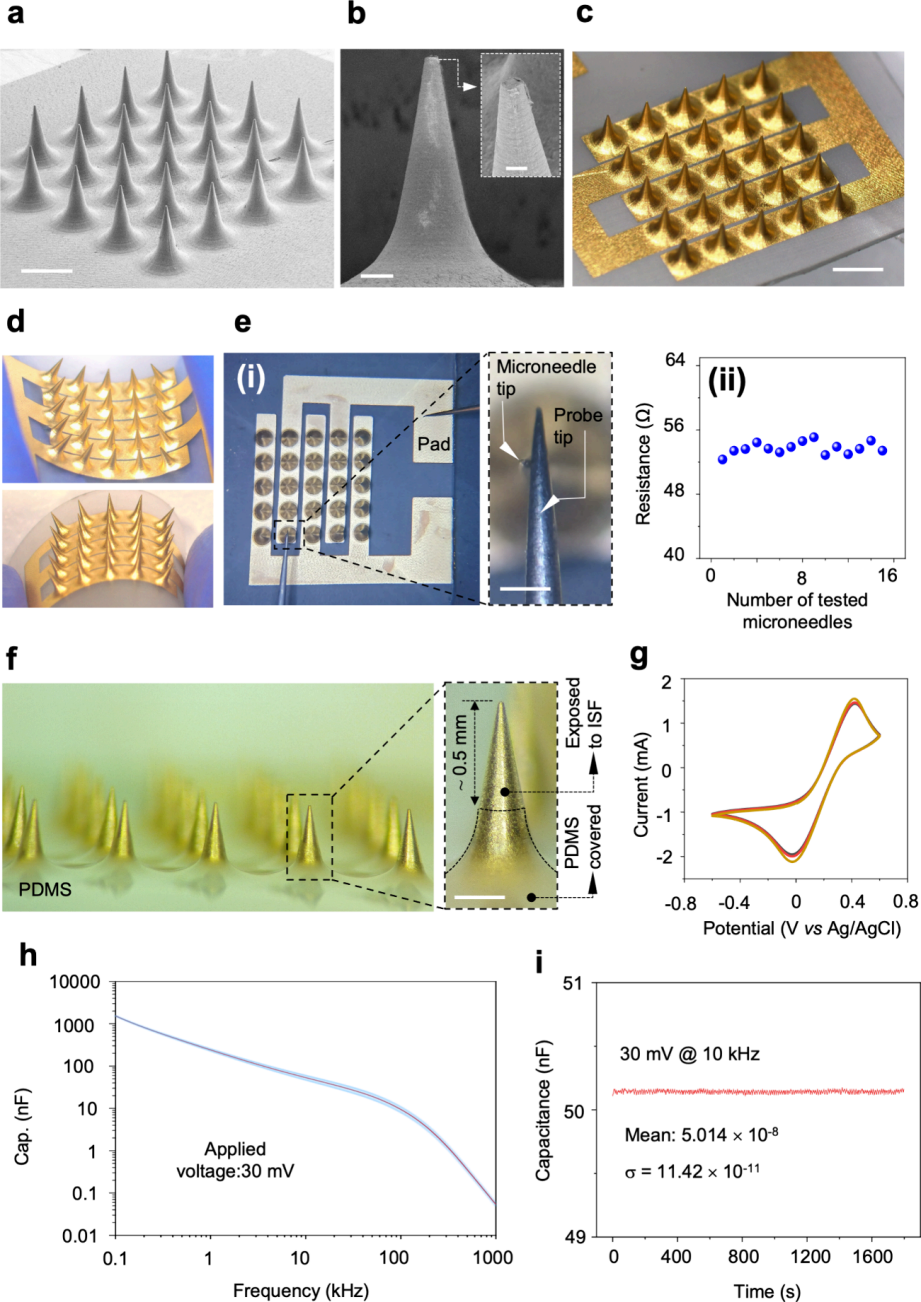
Fabrication and characterization of the MiCaP. (a) SEM image of the fabricated MiCaP (scale bar: 1 mm). (b) SEM image of a single microneedle (scale bar: 100 μm); the inset shows a magnified view of the microneedle tip (scale bar: 60 μm). (c) Optical image of MiCaP showing the IDE design with five fingers, each covering a row of five microneedles (scale bar: 2 mm). (d) Optical images demonstrating the mechanical flexibility of the MiCaP. (e) Optical image showing the setup for electrical resistance measurement from the microneedle tip to the contact pad (i) and summary of resistance measurements from 16 different microneedles (ii). (f) Optical images of MiCaP with the base covered with PDMS (left) and a single microneedle highlighting the exposed area of a microneedle to ISF (scale bar: 300 μm) (right). (g) CV of three independently fabricated MiCaP sensors, demonstrating consistent electrochemical performance. (h) EIS results of three MiCaP sensors, showing reproducible capacitance profiles across the 0.1–1000 kHz frequency range. (i) Result of continuous capacitance measurement of MiCaP exposed to 0.1 × PBS for 1800 s.

In addition to electrical and electrochemical evaluation, the mechanical performance of the device has been tested for its capability to effectively pierce the epidermis and reach the dermis layer of the skin. Figure 3a shows a SEM image of rat skin treated with MiCaP, highlighting an array of indentations caused by MiCaP microneedles, where a zoomed-in view of a single indentation is shown in Figure 3b. The microneedle insertion profile was also inspected by optical microscopy on control and MiCaP-treated rat skins, as shown in Figure 3c, indicating proper insertion. The penetration profile was further examined using a confocal microscope, as shown in Figure 3d, revealing a penetration depth of more than 0.7 mm, which is enough for reaching the dermis layer of the skin. A video of the insertion profile of one microneedle is shown in Video S2. Additionally, SEM images of MiCaP after the penetration test demonstrate that the microneedles maintained their original shape (Figure 3e), confirming their mechanical integrity and their capability of skin penetration without breakage.

**Figure 3:**
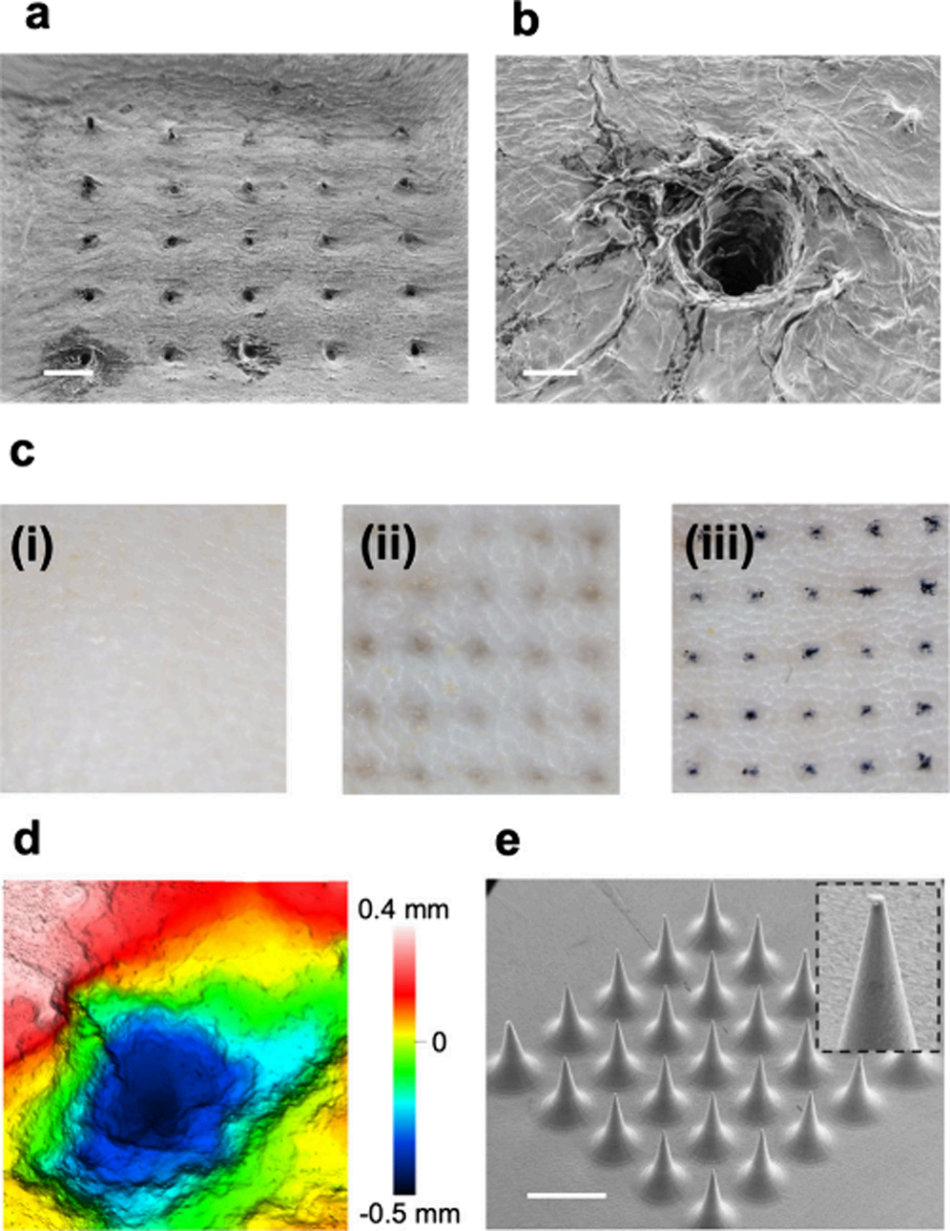
Performance characterization of the MiCaP in skin penetration. (a) SEM image showing indents created in rat skin after administration by the MiCaP (scale bar: 1 mm). (b) A zoomed-in view of an indent (scale bar: 100 μm). (c) Optical images for MiCaP insertion profile. Shaved and cleaned untreated rat skin, serving as the control (i), rat skin after treatment with MiCaP for 15 min. The visible indentations indicate successful microneedle insertion into the skin (ii), trypan blue spots within the rat skin resulting from the insertion of MiCaP stained with trypan blue (iii). The stained regions confirm the effective penetration of the microneedles into the skin. (d) Confocal microscopy image showing a penetration depth of at least 0.7 mm into rat skin. (e) SEM images of the MiCaP after skin penetration experiment (scale bar: 2 mm).

### Sensing Mechanism and Electrical Characterization

#### Sensing Mechanism

The detection principle of the MiCaP relies on monitoring changes in the interfacial capacitance (*C*_int_) resulting from target binding events at the microneedle surface. For a bare microneedle, *C*_int_ arises from the electric double layer (EDL) when it is exposed to the solution. The EDL naturally forms at the microneedle–solution interface, which can be modeled as $C_{\mathrm{int}}=\varepsilon_{s}\varepsilon_{0}\frac{A}{d_{1}}$ (Figure 4a,i), where ε_s_ is the dielectric constant of the solution, ε_0_ is the vacuum permittivity, *A* is the microneedle surface area, and *d*_1_ is the EDL thickness.^45,46^ Functionalizing the microneedle surface with a specific cTnI capture antibody introduces an additional dielectric layer of *d*_2_ that effectively increases the thickness of EDL to *d*_1_ + *d*_2_ (Figure 4a,ii), where the interfacial capacitance of the microneedle after antibody functionalization becomes

1 $$C_{\mathrm{int\_functionalized}}=\frac{\varepsilon_{0}\varepsilon_{s}\varepsilon_{a}A}{{\varepsilon_{s}d}_{2}+{\varepsilon_{a}d}_{1}}$$

where ε_*a*_ is the dielectric constant of the antibody and *d*_2_ is related to the antibody size. Upon binding of cTnI to antibody, the EDL thickness increases further and becomes *d*_1_ + *d*_2_ + *d*_3_ (Figure 4a,iii), where the interfacial capacitance becomes

2 $$C_{\mathrm{int\_binding}}=\frac{\varepsilon_{0}^{2}\varepsilon_{s}\varepsilon_{a}\varepsilon_{t}A^{2}}{{\varepsilon_{s}d}_{2}d_{3}+{\varepsilon_{a}d}_{1}d_{3}+{\varepsilon_{t}d}_{1}d_{2}}$$

where ε_*t*_ is the dielectric constant of the cTnI and *d*_3_ is related to the cTnI size. Based on the extracted equations for the interfacial capacitance of the microneedles before (eq 1) and after (eq 2) cTnI binding to the antibody, the concentration of cTnI can be quantified by the calculation of the normalized capacitance change according to the following equation:

3 $$\%\frac{C_{\mathrm{int\_binding}}-C_{\mathrm{int\_functionalized}}}{C_{\mathrm{int\_functionalized}}}=\left(\frac{\varepsilon_{0}\varepsilon_{t}A\left(\varepsilon_{s}d_{2}+\varepsilon_{a}d_{1}\right)-\left(\varepsilon_{s}d_{2}d_{3}+\varepsilon_{a}d_{1}d_{3}+\varepsilon_{t}d_{1}d_{2}\right)}{\varepsilon_{s}d_{2}d_{3}+\varepsilon_{a}d_{1}d_{3}+\varepsilon_{t}d_{1}d_{2}}\right)\times 100$$

**Figure 4:**
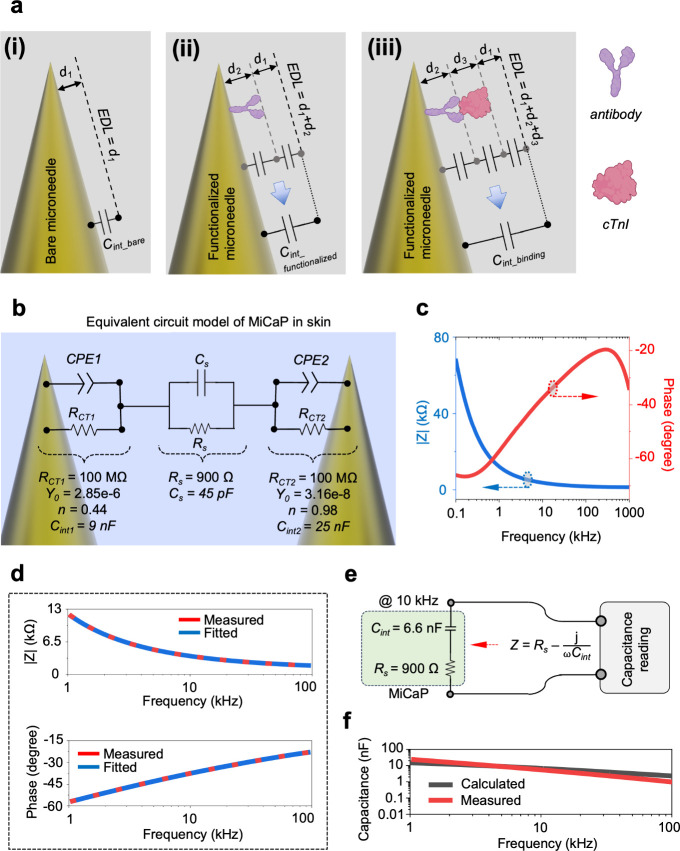
Electrical characterization of MiCaP. (a) Changes in EDL thickness at different stages of MiCaP functionalization and cTnI detection; in the bare sensor the EDL thickness is *d*_1_ (i), then the sensor is functionalized with antibody and the thickness of the EDL is *d*_1_ + *d*_2_ (ii), after binding of cTnI molecules to the antibody the thickness of the EDL becomes *d*_1_ + *d*_2_ + *d*_3_ (iii). (b) The equivalent circuit model of MiCaP when inserted in rat skin. (c) Measured impedance profile of the MiCaP (inserted in rat skin), used for curve fitting and circuit parameters extraction. (d) Plot of measured and fitted impedance data of the MiCaP for the frequency range of 1 to 100 kHz, indicating the accuracy of the fitting. (e) The simplified two-element equivalent circuit model of the MiCaP. (f) Measured and calculated *C*_EDL_ for the frequency range of 1 to 100 kHz.

This nonfaradaic detection method offers a label-free approach to monitor biomolecular interactions by measuring only the capacitance of the sensor. The calculated percentage (*C*_int_binding_ – *C*_int_functionalized_/*C*_int_functionalized_) is used as the output metric of the sensor, as using a normalized capacitance change instead of absolute capacitance enhances the repeatability of the results.

#### Equivalent Circuit Model

When the MiCaP is immersed in an electrolytic solution, neighboring microneedles can be approximated by an equivalent circuit model, as shown in Figure 4b, in which the constant phase element (CPE) belongs to interfacial capacitance, *R*_ct_ is the charge transfer resistance, *R*_s_ is the electrolyte resistance, and *C*_s_ is the electrolyte capacitance. To extract the equivalent circuit model parameters, MiCaP was inserted into rat skin, and its impedance profile was measured using the impedance analyzer, as shown in Figure 4c. By performing curve fitting of the measured impedance data over the frequency range of 1 to 100 kHz with the equivalent circuit model, the circuit parameters were extracted, as illustrated in Figure 4b. Then, eq 4 has been used to convert CPE into *C*_int_.^47^

4 $$C_{\mathrm{int}}=\frac{Y_{0}{\left(\omega \right)}^{n-1}}{\sin\left(n\pi /2\right)}$$

where *Y*_0_ and *n* are the characteristic parameters of the CPE, and ω is the angular frequency. Solving this equation at the frequency of interest (*f* = 10 kHz) for CPE1 and CPE2 in the equivalent circuit model gives the values for *C*_int1_ and *C*_int2_ as 9 and 25 nF, respectively. The variation in capacitance values (9 vs 25 nF) is primarily due to asymmetries introduced during fabrication and surface treatment. Differences in the PDMS insulation layer, surface texture, surrounding dielectric properties, and functionalization can alter local electrochemical responses. Consequently, the measured capacitance reflects the overall interfacial characteristics rather than uniform behavior across the entire array (for more information, read Supplementary Note (SN) 1). Based on the extracted values, the impedance of *C*_int1_ and *C*_int2_ at the frequency of 10 kHz is calculated (using 1/(ω*C*_int1_) and 1/(ω*C*_int2_)) to be 1.8 and 637 Ω, respectively. Considering the parallel connection of these capacitances with *R*_ct1_ and *R*_ct2_ (which have an impedance of 100 MΩ), these resistors can be neglected from the equivalent circuit model. Doing the same calculations for *R*_s_ and *C*_s_ at 10 kHz, the impedance of the *C*_s_ (1/ω*C*_s_) becomes 353 kΩ, which can be disregarded concerning the impedance of the *R*_s_ (900 Ω), as they are connected in parallel. Based on these simplifications, the final equivalent circuit model of the MiCaP can be considered as a series connection of *C*_int1_, *C*_int2_, and *R*_s_, where *C*_int1_ and *C*_int2_ can be further simplified as they are connected in series. The final equivalent circuit model of the MiCaP can be considered as the series connection of *C*_int_ and *R*_s_ (Figure 4e), where *C*_int_ = *C*_int1_ × *C*_int2_/(*C*_int1_ + *C*_int2_).

Based on the simplified equivalent circuit model (Figure 4e), the *C*_int_ of MiCaP at 10 kHz was calculated to be approximately 6.6 nF. This value agrees with the experimentally measured capacitance of the skin-inserted MiCaP device, which was 5.6 nF at the same frequency. Although the discussion of circuit simplification focused on 10 kHz, the approach remains valid across a broader frequency range from 1 to 100 kHz. As shown in Figure 4f, the measured and calculated *C*_int_ values exhibit close agreement across the tested frequency range, supporting the validity of the proposed two-element equivalent circuit model. An important implication of this simplified model is that the capacitance measured from MiCaP corresponds predominantly to the *C*_int_ component, which provides information about molecular binding at the microneedle surface. Therefore, monitoring MiCaP capacitance variations over time using an impedance analyzer or a capacitance measurement system enables real-time tracking of molecular interactions or deposition events at the surface of the functionalized microneedles within the dermal layer. This nonfaradaic sensing approach offers significant benefits for wearable biosensing applications, including straightforward implementation, system integration, and fast signal acquisition. By avoiding the use of redox-active components, it minimizes signal interference and enables a more streamlined and reliable sensor architecture.

### MiCaP Biosensor Development and Validation

To make use of MiCaP as a biosensor for cTnI detection, its surface was functionalized. The MiCaP was cleaned by immersing it in ethanol for 3 h, rinsed with DI water, dried with nitrogen gas, and treated with a plasma cleaner for 5 min. Then, a solution of cysteamine hydrochloride prepared in ethanol was loaded onto the MiCaP and allowed to react overnight in a specific chamber (Figure S2) at room temperature. The MiCaP was then rinsed with pure ethanol and blown with nitrogen gas to remove unbound molecules, resulting in an amine-functionalized surface. Next, a solution of 5% glutaraldehyde in deionized water was loaded into the chamber containing the MiCaP with the amine-functionalized surface for two h to introduce an aldehyde-terminated surface. The MiCaP was removed from the chamber and rinsed with running deionized water. To covalently bind the mAbs to the MiCaP surface, a 100 ng/mL mAbs solution in 0.1 × PBS was added to the MiCaP chamber and then incubated for three h at room temperature. The remaining mAbs residues were washed out using 0.1 × PBS (details of surface functionalization chemistry is provided in Supplementary Note 2). Finally, to minimize nonspecific protein adsorption and passivate the unmodified gold regions, the sensor surface was treated with 1 mM 6-mercapto-1-hexanol (MCH) in DI water for 2 h. MCH binds to the remaining bare gold, forming a compact blocking layer.

The AFM images of the MiCaP surface before and after antibody immobilization are shown in Figure 5a, indicating proper surface functionalization. The bare gold surface showed a relatively smooth profile, with height variations reaching approximately 10.5 nm. In contrast, the fully functionalized surface displayed increased heterogeneity with variations up to about 17.0 nm. This increase in surface roughness is attributed to the added nanostructure introduced by the antibody layer (details are provided in Supplementary Note 3). Furthermore, EIS was performed in 5 mM K_3_[Fe­(CN)_6_] in PBS with pH of 7.4 at the frequency range of 0.1 Hz to 100 kHz by utilizing external reference (Ag/AgCl) and counter (platinum) electrodes to measure the impedance variation after each surface functionalization step (experimental details are provided in Supplementary Note 4). The EIS measurements represented in Figure 5b show step-by-step modification of the bare MiCaP starting with the cleaned Au-coated microneedles (i), immobilization of cTnI-specific antibody (ii), blocking residual sites with MCH (iii), and binding of cTnI to antibody (iv). The results show that upon incubation of MiCaP with antibody, the linear response from the bare MiCaP changed to a semicircle with a drastic increase of *R*_ct_ to 3.7 kΩ as the antibody deposition obstructed the charge transfer process of redox species. After MCH deposition, the *R*_ct_ value further increased to 4.4 kΩ as a result of blocking of the unoccupied areas of the microneedle. Furthermore, in the last step (iv), binding of cTnI to the immobilized antibody was demonstrated by incubating a MiCaP in 100 pg/mL cTnI, which resulted in a further increase in *R*_ct_ value to 6.1 kΩ, which is an indication of successful cTnI probe interaction. The EIS data were fitted and analyzed using a standard Randles equivalent circuit model. To demonstrate the reproducibility and robustness of the modification process, the EIS experiment was repeated on three independent randomly selected MiCaP. Also, it is worth mentioning that, since EIS characterization of the MiCaP was conducted in redox active solution, the faradaic impedance spectra were obtained from the Nyquist results, which involve both the semicircle and the inclined straight line.

**Figure 5:**
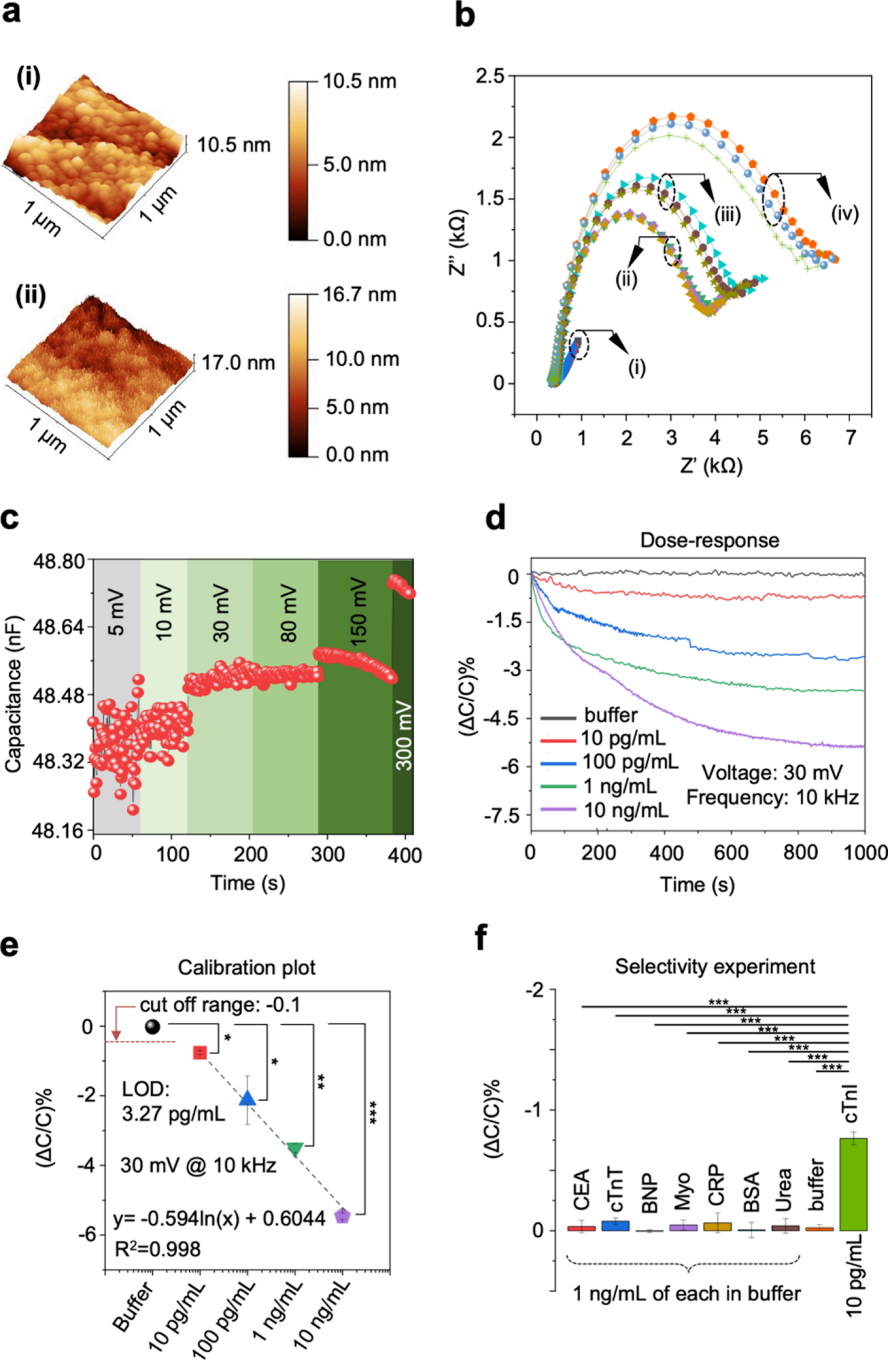
MiCaP biosensors development and characterization. (a) AFM images of the MiCaP before (i), and after antibody immobilization (ii). (b) EIS results indicate an increase in the *R*_ct_ due to the absorption of anti-cTnI and MCH to MiCaP and the binding of 100 pg/mL of cTnI to the antibody. (c) MiCaP capacitance interrogation signal amplitude selection. For low amplitudes (<30 mV) of the excitation signal, the measured capacitance fluctuates and is not reliable. The capacitance became stable by increasing the signal to 30 and 50 mV. However, for higher signal levels (>100 mV), the applied signal triggers the EDL and leads to unreliable capacitance change in the sensor output. (d) Dose–response of the MiCaP for various concentrations of cTnI. (e) Calibration curve and calibration equation of the MiCaP. Each dose was repeated at least three times on a new functionalized sensor. Error bars indicate standard deviation. (f) Selectivity experiment of the MiCaP concerning seven different interfering molecules spiked in the buffer. Data were expressed as the mean ± SD; *P < 0.05, **P < 0.005, ***P < 0.001.

### In Vitro Capacitive Biosensing with MiCaP

Following the validation of surface functionalization and cTnI binding, the performance of the MiCaP sensor was evaluated through a series of in vitro experiments, including dose–response measurements, calibration curve extraction, and selectivity testing. The capacitance-based signal transduction mechanism of MiCaP is based on the principles outlined previously and supported by experimental observations and theoretical analysis. Capacitance measurements were performed using an impedance analyzer at the frequency of 10 kHz and an excitation amplitude of 30 mV, which provided stable signal acquisition (Figure 5c). During measurements, MiCaP was placed inside a test chamber while connected to the impedance analyzer (Figure S2). To determine the dose–response, MiCaP was exposed to four different concentrations of cTnI (10 pg/mL, 100 pg/mL, 1 ng/mL, and 10 ng/mL) prepared in 0.1 × PBS. Each concentration, along with a blank buffer control, was tested at least three times. As shown in Figure 5d, after applying 200 μL of each sample onto the sensor, the capacitance response decreased progressively over time and stabilized after approximately 15 min. The observed normalized capacitance changes (%ΔC/C) were approximately −0.7%, −1.14%, −3.3%, and −5.4% for 10 pg/mL, 100 pg/mL, 1 ng/mL, and 10 ng/mL of cTnI, respectively. This decrease in capacitance is attributed to the binding of cTnI molecules to the immobilized probes, in agreement with previous studies on capacitive biosensing mechanisms.^48,49^ Based on these dose–response data, a calibration curve was constructed (Figure 5e). The fitted calibration equation was determined to be *y* = −0.594 ln­(*x*) + 0.6044, with a high coefficient of determination (*R* ^2^ = 0.998), indicating good linearity in the tested concentration range. The sensor’s cutoff value was calculated as μ + (3 × σ) = −0.1%, where μ (−0.02448%) and σ (0.02523%) represent the mean and standard deviation of MiCaP’s response to blank buffer, respectively. The corresponding LOD was estimated to be 3.27 pg/mL by using the calibration curve. Potential interfering proteins were tested at concentrations of 1 ng/mL to assess the selectivity of MiCaP. These included carcinoembryonic antigen (CEA), cardiac troponin T (cTnT), C-reactive protein (CRP), NT-proBNP, Myoglobin, BSA, and urea. As shown in Figures 5f and S4, the responses obtained for these interfering molecules remained within the cutoff range, indicating minimal cross-reactivity and supporting the specificity of MiCaP toward cTnI detection.

### Spike and Recovery Test

A spike-recovery test was conducted to evaluate the performance of MiCaP in detecting cTnI within a complex biological matrix. Commercial cTnI-free human serum was used for the experiment. To reduce matrix effects inherent to serum, serum was diluted 1:1 with PBS before testing. The diluted serum was first applied to MiCaP to determine its baseline cTnI level. This measurement was performed using three independently functionalized fresh sensors, and the average sensor response was −0.68%, corresponding to a cTnI concentration of 8.64 pg/mL, as determined by the calibration curve. Next, the same serum samples were spiked with known concentrations of cTnI at 15, 800, and 2000 pg/mL. Each spiked sample was tested in triplicate on freshly prepared and functionalized MiCaP devices. The average sensor responses for the 15, 800, and 2000 pg/mL spiked samples were −1.25%, −3.33%, and −3.89%, respectively, corresponding to calculated concentrations of 21.15, 754.23, and 1934.75 pg/mL. Based on these results, the percentage recovery (%*R* = ((*A* – *B*)/*S*) × 100, where *A* is the sensor response after spiking, *B* is the sensor response before spiking, and *S* is the spiked value) of the MiCaP was calculated and summarized in Table 1, which indicates a recovery rate of higher than 93% for all samples. These results underscore MiCaP’s reliability in detecting and quantifying cTnI in complex biological matrices, supporting its potential for in vivo experiments.

**Table 1:** Spike and Recovery Test Results

	spike level (pg/mL)	measured by MiCaP (pg/mL)	% recovery
cTnI-free human serum (1:1 dilution)	0	8.64	NA
	15	22.65	93.4
	800	754.23	93.19
	2000	1934.75	96.3

### Animal Model and In Vivo Analysis

There is growing interest in minimally invasive monitoring of cTnI from ISF, however, its presence and concentration in ISF remain underreported in the literature.^23^ To address this gap and support the validity of our in vivo biosensing experiments, we first performed an independent experiment for the assessment of cTnI levels in ISF with the aid of a standard clinical immunoassay system (DXI 600, Beckman Coulter) and compared them with serum samples. The experimental procedure and corresponding results are provided in Figure S5. Baseline cTnI concentrations were observed in ISF and serum of healthy rats.^50^ Although variations were noted between the two compartments, no statistically significant difference was found under stable physiological conditions. These results confirm the presence of cTnI in ISF and highlight its potential as a viable biofluid for minimally invasive cardiac biomarker monitoring. Building on this finding, we conducted in vivo experiments to evaluate the performance of MiCaP for real-time monitoring of cTnI levels directly in the dermal ISF, as a representative minimally invasive and POC-compatible diagnostic platform.

The methodology for the animal experiments is illustrated in Figure 6a. Two groups of Wistar albino rats (6–8 weeks old), each consisting of three animals, were used for the in vivo studies—one as the experimental group and the other as the control group. A benchtop setup was employed for real-time capacitance measurements using the impedance analyzer, alongside a heating pad to maintain the rats’ body temperature during anesthesia (Figure 6b). The MiCaP was affixed onto the shaved and cleaned dorsal skin by using double-sided medical adhesive. For the experimental group, a dose of 200 ng/kg of cTnI spiked in physiological saline was administered via tail vein injection, followed by continuous capacitance monitoring over 40 min. The initial capacitance of MiCaP was approximately 17.5 nF, which began to decrease about 10 min postinjection and stabilized after approximately 35 min at around 16.9 nF. This temporal delay is consistent with the expected pharmacokinetics of intravenously administered biomarkers reaching ISF compartments, as reported previously in the literature.^51^ The calculated sensor output (%ΔC/C) showed a change of approximately −3.2% (Figure 6c). To confirm the specificity of the MiCaP response to cTnI, control experiments were conducted wherein blank physiological saline (without cTnI) was injected into the tail vein of control animals under identical conditions. As shown in Figure 6d, the sensor exhibited minimal change (%ΔC/C of −0.14%), indicating negligible nonspecific response and supporting the specificity of the sensor toward cTnI. Using the previously established calibration curve, the %ΔC/C values were converted into cTnI concentrations as 3.5 pg/mL for the control animal and 604 pg/mL for the experimental animal. Subsequently, blood samples were collected from the tail vein, serum was extracted by centrifugation, and cTnI levels were quantified using a standard clinical immunoassay system (DXI 600, Beckman Coulter). The serum cTnI concentrations were found to be 4.7 pg/mL for the control and 3100 pg/mL for the experimental animal. The in vivo experiments were independently repeated two additional times using the same protocol to evaluate reproducibility. The %ΔC/C values and corresponding ISF cTnI concentrations from all six experiments (three control and three experimental animals) are summarized in Figure 6e–f. Also, the corresponding serum cTnI values are shown in Figure 6f for comparison. The control group results demonstrated good agreement between ISF and serum cTnI concentrations, aligning with previously reported baseline levels in healthy rats in the literature.^50^ Importantly, statistical analysis revealed no significant difference between the ISF and serum cTnI concentrations in the control group, indicating that the sensor accurately reflects physiological cTnI levels under baseline conditions. At stable systemic cTnI levels, serum and ISF concentrations tend to match closely because cTnI can equilibrate across capillaries over time, binding effects in the extracellular matrix are minimal under these conditions, and the relatively slow turnover of dermal ISF helps maintain steady-state levels — all contributing to the small deviations typically observed between ISF and serum measurements.^22^ Moreover, it is well documented that under stable systemic conditions, the ratio of biomarker concentrations in ISF relative to serum depends on molecular weight.^52,53^ For a protein such as cTnI (∼25 kDa), theoretical models and experimental data indicate that a concentration ratio of about 50% between ISF and serum exists under equilibrium conditions.^23,54–56^ This level of concentration difference is in good agreement with our observations of cTnI in ISF and serum in the control experiments. In the experimental group, significantly elevated cTnI concentrations were detected in both ISF and serum. As summarized in Figure 6g, the average cTnI concentrations measured in ISF for both control and experimental groups are consistent with those obtained from serum, supporting the sensor’s validity across a wide dynamic range. In the experimental group, the cTnI concentration measured in serum was approximately five times higher than that in ISF. This difference in absolute concentrations can be attributed to the inherent physiological disparity between blood and ISF. It is well documented in the literature that while biomarker concentrations in ISF and serum typically show close agreement under stable physiological conditions, larger deviations are expected under stimulated or pathological conditions. First, cTnI (∼25 kDa) moves relatively slowly from blood into dermal ISF, and under rapid rises in serum levels, ISF concentrations lag due to transcapillary transport delays.^57,58^ Second, cTnI binds to extracellular matrix components like collagen and glycosaminoglycans in the dermis, and as circulating levels increase, these binding sites can become saturated, limiting the amount of free cTnI in ISF.^23^ Third, tissue-resident cTnI may undergo post-translational modifications or partial degradation, resulting in molecular forms that differ from those detected in serum using standard immunoassays.^59^ These effects have been previously reported for other biomarkers as well (e.g., IL-6) and are consistent with the dynamics of ISF.^22^ Furthermore, methodological differences between the measurement approaches are also effective. Specifically, the MiCaP calibration curve was generated by exposing the functionalized device to known concentrations of cTnI prepared in a standard dilution buffer. In contrast, in situ analyte detection in dermal tissue involves a complex, dense matrix that limits diffusion and leads to slower binding kinetics, potentially resulting in a lower concentration at the sensor interface. Additionally, variability in protein distribution across body fluids may contribute to the observed differences.^23^ Furthermore, variations in cTnI levels across animals despite similar cTnI dosing could stem from inter- and intrasubject variability in physiological parameters such as renal clearance, body weight, hydration status, and metabolic rate.^32^ Despite these factors, the cTnI concentrations determined using MiCaP demonstrated strong qualitative agreement with serum measurements (Figure 6f,g). Importantly, MiCaP reliably distinguished between physiological and elevated cTnI states, measuring average concentrations of 3.2 ± 0.4 pg/mL in control animals and 912 ± 683 pg/mL in the experimental group (mean ± SD; Figure 6g). These in vivo results underscore the sensor’s responsiveness to systemic cTnI elevations and demonstrate its suitability for continuous monitoring of cardiac biomarkers in ISF, advancing its potential for wearable and POC diagnostic applications.

**Figure 6:**
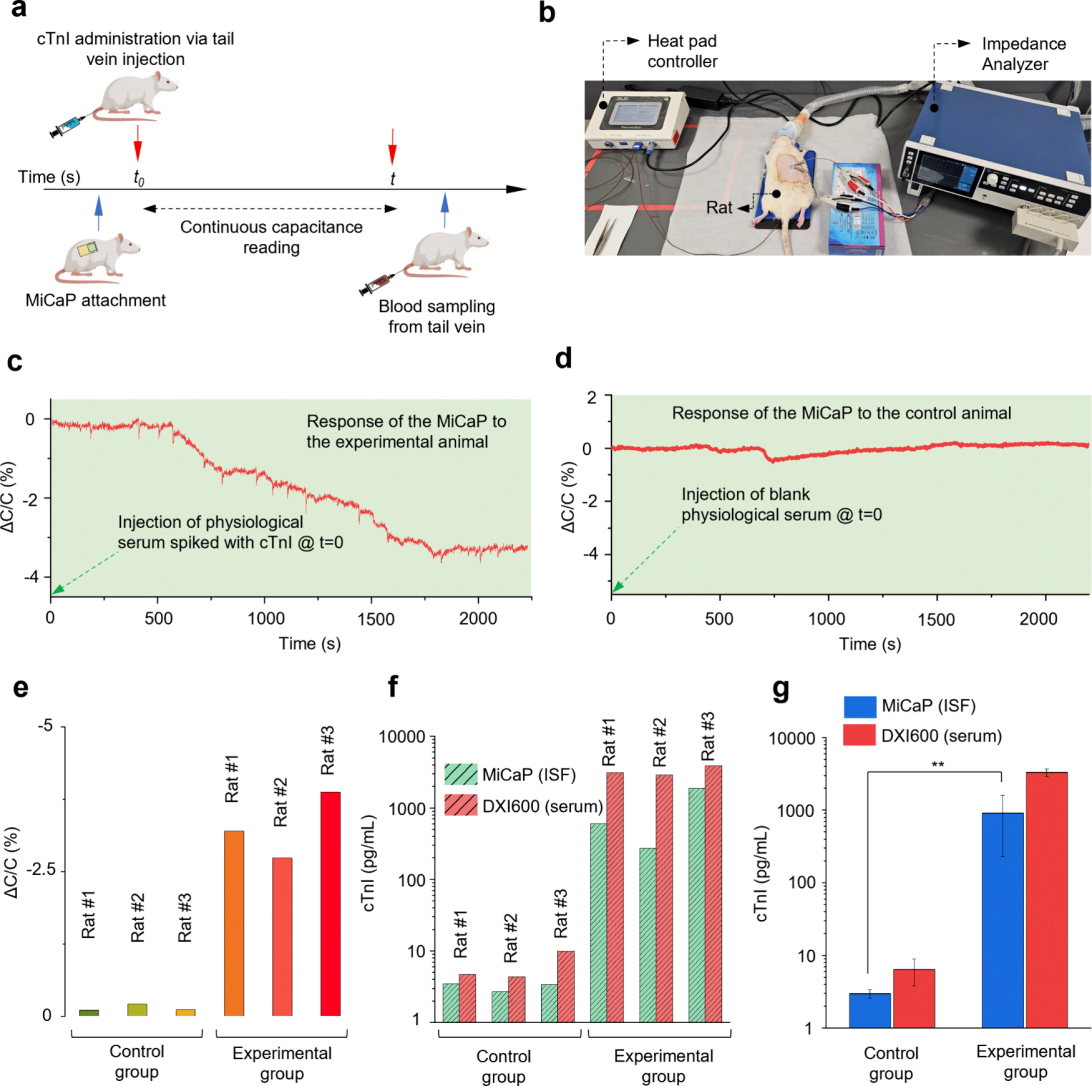
In vivo evaluation of MiCaP for cTnI detection in rat model. (a) Schematic overview of the in vivo experimental procedure. (b) Photograph of the experimental setup showing MiCaP attachment to the rat dorsal skin and real-time capacitance monitoring using the impedance analyzer. (c) MiCaP response (%Δ*C*/*C*) during in vivo testing in an experimental animal following cTnI administration. (d) MiCaP response (%Δ*C*/*C*) during in vivo testing in an control animal injected with blank physiological saline solution. (e) Summary of %Δ*C*/*C* values obtained from six rats (three experimental and three control animals). (f) Comparison of concentrations of cTnI measured in serum and ISF in the control group (left) and the experimental group (right) (*n* = 3 per group). cTnI in dermal ISF determined by MiCaP (green) exhibited a good qualitative correlation with that in serum tested by DXI 600 (red). (g) Summary of ISF and serum cTnI concentrations measured by MiCaP and DXI 600 across all six rats. Data are presented as mean ± SD. Statistical significance determined at **P < 0.005.

## Conclusion

In this work, we developed a wearable microneedle patch to monitor cTnI in ISF. The patch comprises 5 × 5 conical microneedle arrays covered with interdigitated electrodes (Cr/Au) for reliable EDL capacitance measurements. Characterization studies of the MiCaP demonstrated its potential for cTnI sensing, with a dynamic range of 10 pg/mL to 10 ng/mL, a LOD of 3.27 pg/mL, and a response time of less than 15 min. Additionally, the selectivity of the patch was confirmed through experiments in buffer, where its performance was evaluated against seven strong interfering molecules, showing no significant interference. We further validated the in vivo application of the MiCaP by attaching it to the dorsal skin of a rat to monitor real-time changes in the cTnI concentration in the ISF. The in vivo results were validated by the cTnI concentration in serum. Establishing a correlation plot between cTnI levels in ISF and serum will be an important step for future clinical applications. In this study, the primary focus was on the development and validation of a biosensing platform for direct cTnI detection in ISF, while calibration with serum levels is planned as part of future clinical research. This study marks a significant advancement in the field and presents a clear pathway toward developing next-generation, patient-centered wearable systems for remote monitoring, which holds the potential to advance digital healthcare.

## Materials and Methods

All materials and methods are listed in Supporting Information.

## Supplementary Material







## Notes

Additional experimental details, materials, and methods (PDF)
Video S1: A video of fabricated MiCaP taken by an optical microscope (MP4)
Video S2: A video of a single microneedle insertion into rat skin (MP4)

## §

Bio/CMOS Interfaces (BCI) Laboratory, École Polytechnique Fédérale de Lausanne (EPFL), 2000 Neuchatel, Switzerland
