# Supplementary material for: Minimally Invasive and in Situ Capacitive Sensing of Cardiac Troponin I from Interstitial Fluid
Source: ACS Sens. 2025 Jul 28;10(12):9335–46. doi: 10.1021/acssensors.5c01691 (PMC12751114; doi:10.1021/acssensors.5c01691)
Supplement: Supplementary file 1 [file se5c01691_si_001.pdf]

Minimally invasive and in situ capacitive sensing of cardiac troponin I from interstitial fluid

Hadi Mirzajani[1], Parviz Zolfaghari[1], Beril Yagmur Koca[1], and Hakan Urey*[1,2]*

[1] Department of Electrical and Electronics Engineering, Koç University, 34450 Istanbul, Türkiye

[2] Koç University Research Center for Translational Medicine (KUTTAM), 34450 Istanbul, Türkiye

* *hmirzajani@ku.edu.tr*

* *hurey@ku.edu.tr*

Table of contents:

Table S1. A comparison table of biosensors for cTnI detection reported in the literature.

Table S2. A list of microneedle-based biosensors for in situ biomarker monitoring.

Figure S1. Schematic illustration of the fabrication process flow of MiCaP.

Figure S2. An optical image of the chamber fabricated from polytetrafluoroethylene (PTFE) by an in-house CNC micromachining tool to house the MiCaP during the in vitro experiments.

Supplementary Note 1: Circuit modeling

Supplementary Note 2: Surface functionalization chemistry

Figure S3. Stepwise surface functionalization of the gold-coated MiCaP biosensor for antibody immobilization.

Supplementary Note 3: Atomic force microscopy characterization

Supplementary Note 4: Electrochemical Impedance Spectroscopy (EIS) Protocol

Figure S4. Selectivity experiment results.

Figure S5. Diagnostic potential of ISF for cTnI monitoring

Supplementary Note 5: Materials and Methods

Video S1. A video of fabricated MiCaP taken by an optical microscope.

Video S2. A video of a single microneedle insertion into rat skin.

Table S1. A comparison table of biosensors for cTnI detection reported in the literature.

Ref.	Year	Detection Technique	Output Metric	LOD	Dynamic Range	Detection Time	Detection Medium	In-vivo	System Integration	Wearable/ Implantable
1	2023	EIS	Resistance	4.6 pg/mL	10 pg/mL - 100 ng/mL	46 min	Whole blood (finger prick)	N	N	N
2	2024	CV, DPV	Voltammetric	0.01 ng/mL	0.01–20 ng/mL	15 min	Human serum (50% diluted with PBS)	N	N	N
3	2023	SWV	Current	8.46 pg/mL	10 pg/mL–100 ng/mL	~10 min	Human serum	N	Y	N
4	2023	SPR	Surface plasmon resonance signal changes	0.52 ng/mL	0.78–50 ng/mL	~10 min	PBS	N	N	N
5	2022	ELISA	Fluorescence intensity	0.19 ng/mL	5–180 ng/mL	~17 min	Human serum	N	N	N
6	2021	Distance-based paper analytical device	Color length	0.025 ng/mL	0.025 to 2.5 ng/mL	~15 min	Whole blood	Y	N	N
7	2021	EIS	Resistance	Mouse: 10.91 pg/mL Human: 6.86 pg/mL	NA	5 min	Whole blood	N	N	N
8	2022	SiNW-FET	Current	NA	NA	~3 min	Capillary blood	N	N	N

9	2024	EIS	Capacitance	0.54 pg/mL	0.1–10,000 pg/mL	~15 min	Whole blood (finger prick)	N	Y	N
10	2021	ECL	ECL signal intensity	0.028 pg/mL	0.1 pg/mL–100 ng/mL	NA	Human serum	N	N	N
11	2022	DPV	Current	10 pg/mL	100 pg/mL - 50000 pg/mL	~60 min	Human serum	N	N	N
12	2022	CV, ROP	Current	57.14 fg/mL	1 pg/mL – 1 µg/mL	~60 min	Human serum	N	N	N
13	2023	CL	Light intensity	0.6 µg/L	2–25 µg/L	~10 min	PBS	N	N	N
14	2023	PEC	Photocurrent	2 pg/mL	2 pg/mL–10 ng/mL	~10 min	Human plasma	N	N	N
15	2022	SWV	Current	0.01 pg/mL	0.1 pg/mL–1,000 ng/mL	2 min	Human serum	N	Y	N
16	2022	SWV	Current	0.047 pg/mL	0.04–8 ng/mL	NA	Human serum	N	N	N
17	2022	SWV	Current	2.4 fg/mL	10 fg/mL–0.1 µg/mL	NA	Human serum	N	N	N
18	2022	EGFET	Current	<1 pg/mL	0.01–100 ng/mL	~20 min	PBS	N	N	N
19	2023	CV	Current	Antibody: 1 fM Aptamer: 100 aM	100 aM–100 pM	NA	Human serum	N	N	N
20	2024	EIS	Resistance	3.7 pg/mL	0.0244–25 ng/mL	20 min	PBS	N	N	N
21	2023	SWV	Current	6.59 fM	1 pM–100 nM	10 min	Human serum	N	N	N
22	2024	SWV	Current	9.85 fg/mL	10 fg/mL–100 ng/mL	NA	Human serum	N	N	N
23	2024	DPV	Current	13 fg/mL	0.1 pg/mL–10 ng/mL	15 min	Human serum	N	N	N
24	2024	EIS, CV	Current	76.97 pg/mL	0.1 ng/mL–5 ng/mL	NA	Human serum	N	N	N

25	2023	SAW	Fluorescence intensity	44 pg/mL in PBS, 0.34 ng/mL in human serum	0.2–60 ng/mL	NA	PBS & Human serum	N	N	N
26	2024	PEC	Current	14.42 pg/mL	10 – 200 pg/mL	NA	PBS	N	N	N
27	2022	EGFET	Voltage	0.01 ng/mL	0.01–100 ng/mL	NA	PBS & Human serum	N	N	N
28	2022	SWV	Current	70.0 pg/mL for PCN-RuNPs, 50.0 pg/mL for PCN-NiMoO4 NRs	0.1–10,000 ng/mL	NA	Human serum	N	N	N
29	2021	SWV	Current	1 pg/mL	0.001–200 ng/mL	NA	Serum	N	N	N
30	2021	DPV	Current	1.7 pg/mL	5 pg/mL–10 ng/mL	NA	Human Serum	N	N	N
31	2021	DPV	Current	0.01 ng/mL	0.01–100 ng/mL	NA	Human plasma	N	N	N
32	2021	SWV	Current	0.16 pg/mL	0.001–250 ng/mL	NA	Human serum	N	N	N
33	2021	EIS	Resistance	0.8 ng/mL	1–400 ng/mL	5 min	Mouse serum	N	N	N
34	2021	EIS	Resistance	0.08 ng/mL	0.1–100 ng/mL	5 min	Mouse serum	N	N	N
35	2021	DPV	Current	0.27 pg/mL	0.3 pg/mL–0.2 ng/mL	NA	PBS	N	N	N
36	2023	LSPR	Wavelength shift	108.15 ng/mL	0–1000 ng/mL	NA	PBS	N	N	N
37	2022	SPF	Fluorescence intensity	0.98 ng/mL	3.9–100 ng/mL	30 min	PBS	N	N	N
38	2022	EIS	Resistance	0.055 pg/mL	0.1 pg/mL–10 ng/mL	NA	Human serum	N	N	N
39	2021	DPV	Current	0.58 ng/mL	5–100 ng/mL	5 min	Human serum	N	N	N
40	2024	Raman spectroscopy	Raman signal intensity	1.43 pg/mL	0.01–100 ng/mL	10 min	Human serum	N	N	N

41	2022	PEC	Photocurrent	0.3 pg/mL	0.001–30 ng/mL	3 min	Human serum	N	N	N
42	2024	FRET	Fluorescence intensity	0.012 ng/mL	0.065–1.96 ng/mL	10 min	Human serum	N	N	N
43	2021	DPV	Current	3 pg/mL	0.01–100 ng/mL	3 min	Human serum	N	N	N
44	2024	SERS	Raman signal intensity	0.27 pg/mL	0.001–100 ng/mL	60 min	Human serum	N	N	N
45	2023	Amperometric	Current	1.91 fg/mL	0.001–100 ng/mL	NA	Human serum	N	N	N
46	2023	Colorimetric	Absorbance change	27 pg/mL	0.05–100 ng/mL	5 min	Human serum	N	N	N
47	2021	DPV	Current	0.1 pg/mL	0.1 pg/mL–100 pg/mL	NA	Human serum	N	N	N
This work	2024	Impedimetric	Capacitive	3.27 pg/mL	10 pg/mL – 10000 pg/mL	<15 min	ISF	Y	Y	Y

CL: Chemiluminescence, CV: Cyclic Voltammetry, DPV: Differential Pulse Voltammetry, ECL: Electrochemiluminescence, EIS: Electrochemical Impedance Spectroscopy, ELISA: Enzyme-Linked Immunosorbent Assay, FET: Field-Effect Transistor, FRET: Fluorescence Resonance Energy Transfer, LOD: Limit of detection, PEC: Photoelectrochemical, SAW: Surface Acoustic Wave, SERS: Surface-Enhanced Raman Scattering, SPF: Surface Plasmon Fluorescence, SPR: Surface Plasmon Resonance, SWV: Square Wave Voltammetry

Table S2. A comparison list of microneedle-based biosensors for in situ biomarker monitoring.

Ref.	Microneedle material	Probe molecule	Target biomarker	Detection mechanisms	faradaic or non faradaic	in vivo
48	PU:PEDOT:PSS composite	L-DOPA (chemo-responsive probe)	Tyrosinase (Tyr)	Redox-active enzymatic (oxidation of L-DOPA by Tyr)	Faradaic	No
49	OrmoStamp polymer with Au coating and PL membrane	Glucose oxidase (GOx) or insulin-selective aptamer	Glucose and Insulin	Enzymatic (GOx) for glucose, aptamer-based for insulin	Faradaic	No
50	Methacrylated hyaluronic acid (MeHA) hydrogel	Thiolated redox-tagged aptamers for glucose and lactate	Glucose and Lactate	Redox-labeled aptamer-based sensing	Faradaic	Yes
51	Dopamine-conjugated hyaluronic acid (DA-HA) hydrogel + PEDOT:PSS	DA (redox mediator); HBD enzyme	3-β-hydroxybutyrate (β-HB, a ketone body)	Enzymatic detection with redox-active mediator	Faradaic	Yes
52	Laser-micromachined stainless steel with Au + PEDOT:PSS coating	Ion-selective membranes (no biological probe molecule)	Na+, K+, Ca2+	Potentiometric sensing (non-enzymatic, non-labeled)	Potentiometric	Yes
53	Gold-plated acupuncture needle with AuNP coating embedded in PDMS	Redox-tagged aptamer (e.g., for tobramycin, vancomycin, doxorubicin, thrombin)	Tobramycin, Vancomycin, Doxorubicin, Thrombin	Redox-labeled aptamer-based electrochemical biosensing	Faradaic	Yes
54	Stainless steel with carbon ink and Ag/AgCl coatings	Ion-selective membranes (for pH, Na+, K+, Ca2+, Li+, Cl−)	pH, Na+, K+, Ca2+, Li+, Cl−	Potentiometric ion-selective sensing	potentiometric	Yes
55	PMMA microneedle array with sputtered metal electrodes (Pt, Ag/AgCl)	Glucose oxidase (GOx), Lactate oxidase (LOx), Alcohol oxidase (AOx)	Glucose, Lactate, Alcohol	Enzymatic amperometric (oxidase enzymes)	Faradaic	Yes
56	Stainless steel microneedles on Ecoflex substrate	Glycine oxidase (GLY-Ox)	Glycine (GLY)	Enzymatic (oxidase) with redox mediator (PB)	Faradaic	Yes
57	SU-8 photoresist with Au and CNT layers	NgAgo guided by gDNA on TDNs	Cell-free DNA (cfDNA), RNA	Electrochemical affinity binding (amplification-free, non-enzymatic)	Faradaic	Yes
58	Graphene-coated conductive microneedles on PDMS substrate	dRNP (deactivated Cas9 + sgRNA)	Cell-free DNA (cfDNA): EBV, sepsis-related, kidney transplant-derived	Electrochemical signal change via CRISPR-Cas9 binding (label-free)	Faradaic	Yes
59	Polycaprolactone (PCL)	Hemin	Nitric oxide (NO)	Redox-active (non-enzymatic, hemin-mediated electrochemical)	Faradaic	Yes
60	3D-printed resin microneedles with sputtered Cr/Pt and electrodeposited Au	HBD enzyme + NAD+ + poly-TBO	β-hydroxybutyrate (BHB)	Enzymatic redox with NADH detection (amperometric)	Faradaic	Yes
61	Hollow microneedle array (pyramidal) filled with carbon paste	Tyrosinase enzyme (biocatalytic) and bare carbon (non-enzymatic)	Levodopa (L-Dopa)	Dual-mode: enzymatic amperometric and non-enzymatic voltammetric	Faradaic	No
62	OrmoComp microneedles with recessed microcavities (MCs), Au-coated	Urease	Urea	Enzymatic with potentiometric readout (ammonia sensing via PABA layer)	potentiometric	No
63	Polycarbonate microneedles (injection molded) with Au, Ag coatings	Lactazyme (DET-type lactate enzyme)	Lactate	Enzymatic with direct electron transfer (DET)	Faradaic	Yes
64	Polyurethane microneedles with Au coating	HRP enzyme immobilized in Cs-rGO hydrogel	Hydrogen peroxide (H2O2)	Enzymatic redox via HRP with chronoamperometric readout	Faradaic	Yes
65	24-gauge hollow microneedle with double-sided flexible electrode strip	Glucose oxidase (GOx) with Fc-PEI redox mediator	Glucose	Enzymatic redox (second-generation biosensor)	Faradaic	Yes
66	Polylactic acid (PLA) hollow microneedles	Glucose oxidase (GOD)	Glucose	Enzymatic redox (GOD/PB/Au system)	Faradaic	Yes
67	Stainless steel (SS201) with Au, Pt, Ag/AgCl coatings	Uricase (for UA), Pt (for H2O2), PANI/CNTs (for pH)	Uric acid (UA), hydrogen peroxide (H2O2, as ROS), pH	Enzymatic for UA and ROS, ion-selective (PANI) for pH	Faradaic for UA and H2O2	Yes
68	Stainless steel MNs on silicone rubber substrate	Lactate oxidase (LOx) + Prussian Blue	Lactate	Enzymatic redox (1st generation, LOx-PB system)	Faradaic	Yes
69	Stainless steel MNs embedded in silicone rubber	Ion-selective membranes for pH and carbonate	Carbon dioxide (CO_2, via pH and $CO3^{2-}$ measurement)	Potentiometric ion-selective electrodes (non-enzymatic)	Potentiometric	Yes

- **Figure S1: MiCaP fabrication process**

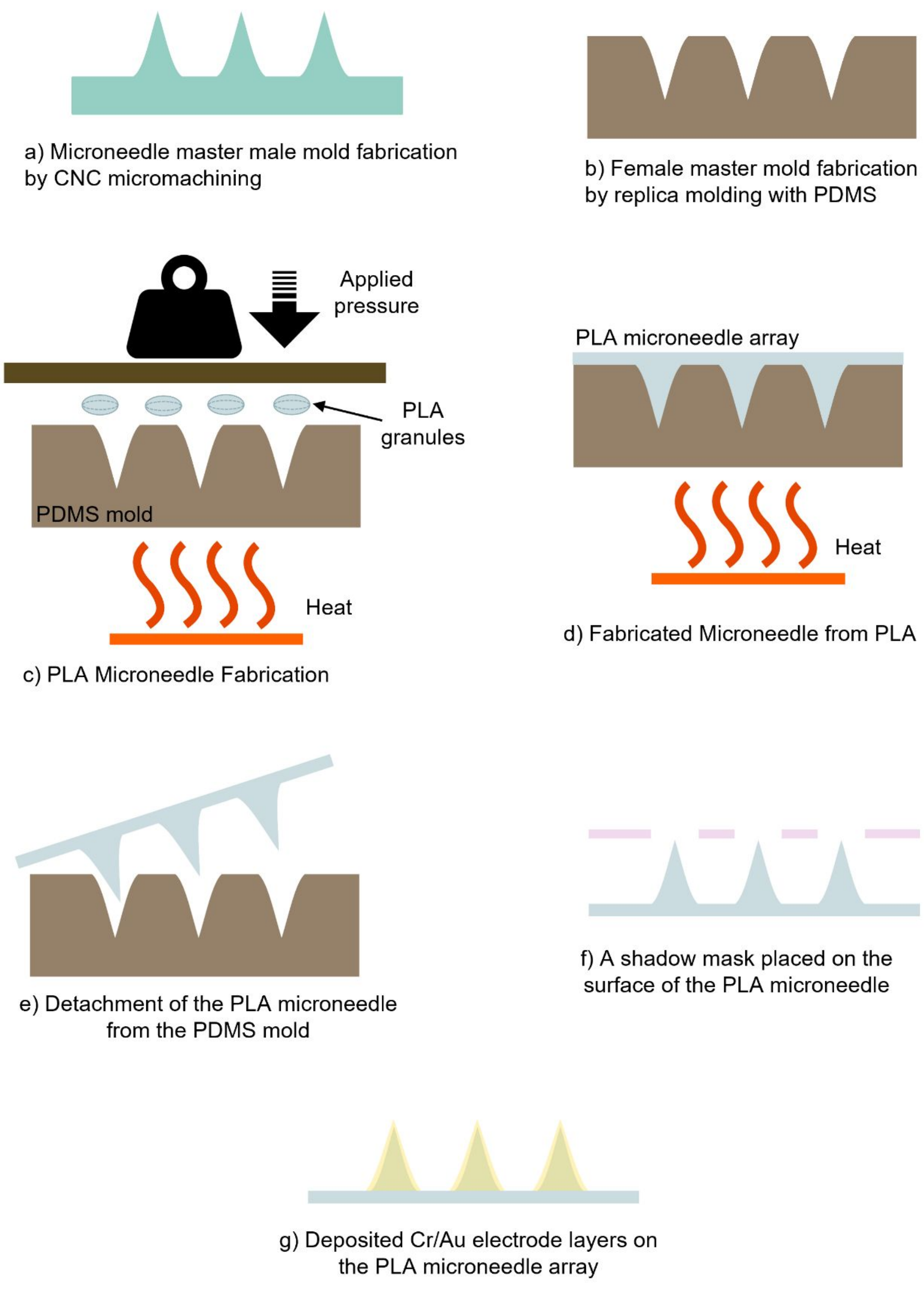

Figure S1. Schematic illustration of the fabrication process flow of MiCaP.

- **Figure S2: Custom-designed measurement chamber**

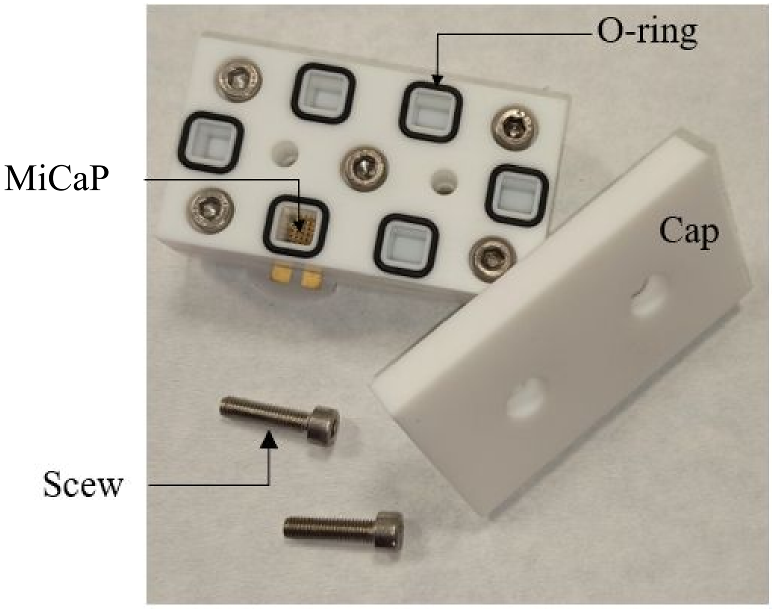

Figure S2. An optical image of the chamber fabricated from polytetrafluoroethylene (PTFE) by an in-house CNC micromachining tool to house the MiCaP during the in vitro experiments.

- **Supplementary Note 1:** Circuit modeling

Although the two capacitances C_{int1} and C_{int2} are associated with microneedles of nominally similar geometry, the observed difference in extracted values (9 nF vs. 25 nF) can be attributed to fabrication- and surface-induced asymmetries. Variability in the deposition of the PDMS insulation layer may lead to differences in the exposed electrode area, thereby affecting the local double-layer capacitance. Additional factors such as variations in surface roughness, dielectric environment, and biomolecular functionalization may also contribute to site-specific electrochemical behavior. As a result, the fitted capacitance values represent effective interfacial responses, rather than idealized or perfectly uniform replicates across the array.

The electrical double-layer (EDL) capacitance of each microneedle in the array is modeled as an independent entity, governed by its geometry, surface functionalization, and biomolecular binding state. Based on classical electrochemical models, including Helmholtz, Gouy-Chapman, and Stern formulations, EDL capacitance is a local interfacial property and is not significantly influenced by the presence of adjacent microneedles under the conditions used in this study. In the equivalent circuit model, the inclusion of a neighboring microneedle element serves as a lumped-parameter abstraction to represent interfacial heterogeneity observed in the impedance spectra. This simplification is not intended to imply physical or electrical coupling between microneedles, nor does it capture the full spatial interaction within the 5×5 array. Rather, it provides a first-order approximation that enables interpretation of the experimental data while preserving computational tractability. To model the C_{EDL} of each microneedle, three classical models can be considered: Helmholtz, Gouy-Chapman, and Stern [70]. Each model provides a framework for understanding the *EDL* at the interface between the microneedle's surface and the surrounding electrolyte (skin, in our case). The Helmholtz model treats the interfacial region as a parallel-plate capacitor and assumes constant charge separation. In this

model, the Helmholtz capacitance (C_{EDL_H}) is defined as Equation S1, with parameters introduced in Table S3 [71].

$$C_{EDL\_H} = \frac{\epsilon_0 \epsilon_r A}{t} \tag{S1}$$

Table S3. The Helmholtz model parameters and related values.

Helmholtz Model Variables	Description
ϵ_0	The permittivity of free space
ϵ_r	Relative permittivity of electrolyte
A	The surface area of a single microneedle. (considering an exposure of about 0.5 mm of the microneedle's tip to the solution)
t	EDL thickness, which is the distance from the electrode to the outer Helmholtz plane (OHP)

While the Helmholtz model simplifies the capacitance as independent of the applied potential, it does not account for the diffuse nature of the ion distribution in real systems. The Gouy-Chapman model extends the Helmholtz concept by incorporating a diffuse layer [72], where ions are distributed according to Boltzmann statistics. In this model, the Gouy-Chapman capacitance (C_{EDL_GC}) [72] is defined as Equation S4, and the parameters are introduced in Table S4.

$$C_{EDL\_GC} = \sqrt{\frac{2z^2 e^2 n_i^0 \epsilon_0 \epsilon_r}{k_B T}} \cosh\left(\frac{ze\phi}{2k_B T}\right), \tag{S2}$$

Table S4. The Gouy-Chapman model parameters and related values.

Gouy-Chapman Model Variables	Description
ϵ_0	The permittivity of free space
ϵ_r	Relative permittivity of electrolyte
e	Electronic charge
k_B	Boltzmann constant
T	Room temperature
ϕ	Applied potential
n_i^0	The number density of i- th ion
z_i	Valence of the i- th ion

The Gouy-Chapman model overestimates the capacitance in practical systems [73]. To address this limitation and enhance accuracy, the Stern model combines both the Helmholtz capacitance and the Gouy-Chapman capacitance in a series configuration by $1/C_{EDL\_S} = \left(1/C_{EDL\_H} + 1/C_{EDL\_GC}\right)$ [73].

Based on the parameters defined in the Helmholtz, Gouy-Chapman, and Stern models, the capacitance of a single microneedle is primarily governed by its own geometry and the properties of the insertion site. As a result, it functions as an independent entity, and the presence of nearby microneedles does not significantly influence its capacitive behavior.

- **Supplementary Note 2:** Surface functionalization chemistry

The MiCaP biosensor surface was chemically functionalized through a sequential process involving gold-thiol bonding, aldehyde–amine crosslinking, and antibody immobilization [74-79].

- Step 1) Formation of Amine-Terminated SAM via Cysteamine:

  Cysteamine hydrochloride ($HS–CH_2–CH_2–NH_2$) was used to modify the gold-coated microneedle surface. The thiol group of cysteamine forms a strong covalent bond with the gold (Au–S), creating a self-assembled monolayer (SAM) with terminal amine ($–NH_2$) groups exposed. This layer serves as the foundation for subsequent functionalization.

- Step 2) activation with Glutaraldehyde:

  The amine-terminated surface was then treated with 5% aqueous glutaraldehyde, a bifunctional molecule containing two aldehyde (–CHO) groups. One –CHO group reacts with the surface $–NH_2$ via Schiff base (imine) formation (–C=N–), anchoring glutaraldehyde to the sensor. The unreacted –CHO group remains available for the next step.

- Step 3) Antibody Immobilization via Amine–Aldehyde Coupling:

  Monoclonal antibodies (mAbs) contain lysine residues with free amine groups. These groups react with the terminal –CHO groups on the surface (also via Schiff base formation), resulting in stable covalent attachment of the antibodies. This process ensures that the antibodies retain their biological activity and orientation on the sensor.

This two-step chemistry forms a stable Schiff base (–C=N–) linkage at each interface and enables robust and specific conjugation of the antibody. Using both $–NH_2$ and –CHO functional groups ensure selective and covalent attachment of antibodies while preserving their orientation and binding capability. Figure S3 shows a schematic illustration of the chemistry used for the surface functionalization. This is a standard approach in biosensor functionalization.

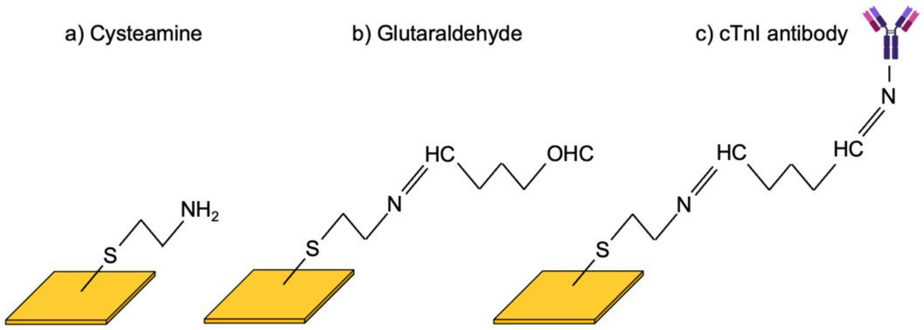

Figure S3. Stepwise surface functionalization of the gold-coated MiCaP biosensor for antibody immobilization. (a) Cysteamine forms a self-assembled monolayer (SAM) on the gold surface via strong Au–S bonds, presenting terminal amine (–NH_2) groups. (b) Glutaraldehyde reacts with surface amines to form Schiff base (–C=N–) linkages, resulting in an aldehyde-terminated interface. (c) Monoclonal antibodies are covalently attached via Schiff base formation between aldehyde groups on the surface and lysine amines on the antibody structure. This two-step functionalization enables stable, oriented, and covalent antibody immobilization on the microneedle surface.

- **Supplementary Note 3:** Atomic force microscopy characterization

Atomic force microscopy (AFM) was used to evaluate changes in the surface morphology of the MiCaP sensor following functionalization. Figure 5 shows 3D AFM height images for (i) the bare gold surface and (ii) the surface after antibody conjugation. The bare gold surface exhibited a relatively smooth topography with height variations of up to ~10.5 nm, while the fully functionalized surface exhibited more heterogeneous features and height variations of up to ~17.0 nm. This increase is attributed to the deposition of antibody layers, which add measurable nanometer-scale topography. The increase in vertical height and surface complexity supports successful biomolecular immobilization, in line with previous AFM-based biosensor studies [80, 81].

- **Supplementary Note 4:** Electrochemical Impedance Spectroscopy (EIS) Protocol

Electrochemical impedance spectroscopy (EIS) was performed to monitor the stepwise surface functionalization of the MiCaP biosensor. All measurements were conducted using a three-electrode setup consisting of the following:

Working electrode: Au-coated MiCaP microneedle array

Reference electrode: Ag/AgCl (3 M KCl)

Counter electrode: Platinum wire

EIS measurements were carried out using an Autolab PGSTAT 101 potentiostat controlled via NOVA 2.1 software. The impedance spectra were recorded in a 5 mM solution of potassium ferricyanide ($K_3[Fe(CN)_6]$) dissolved in 1× PBS, pH 7.4. This redox couple was selected due to its reversible electrochemical behavior and sensitivity to changes in surface charge transfer resistance, making it widely applicable for biosensor characterization. The EIS measurements were performed under the following conditions:

AC amplitude: 10 mV (rms)

DC bias: Open circuit potential (OCP), typically +100 mV vs. Ag/AgCl

Frequency range: 0.1 Hz to 100 kHz

Number of data points per decade: 10

Temperature: Room temperature (~22–23 °C)

Electrolyte volume: 5 mL, contained in a Teflon cell inside a Faraday cage

After each functionalization step (bare gold, antibody immobilization, MCH blocking, and cTnI binding), the MiCaP sensor was gently rinsed with DI water and dried under a nitrogen stream prior to the next measurement. Each measurement was conducted immediately after sample preparation to minimize variability. The Nyquist plots were analyzed using a standard Randles equivalent circuit, and the R_{ct} parameter was used as the main indicator to track changes in surface interfacial properties. Curve fitting was performed using ZVIEW software.

- **Figure S4**. Selectivity experiment results.

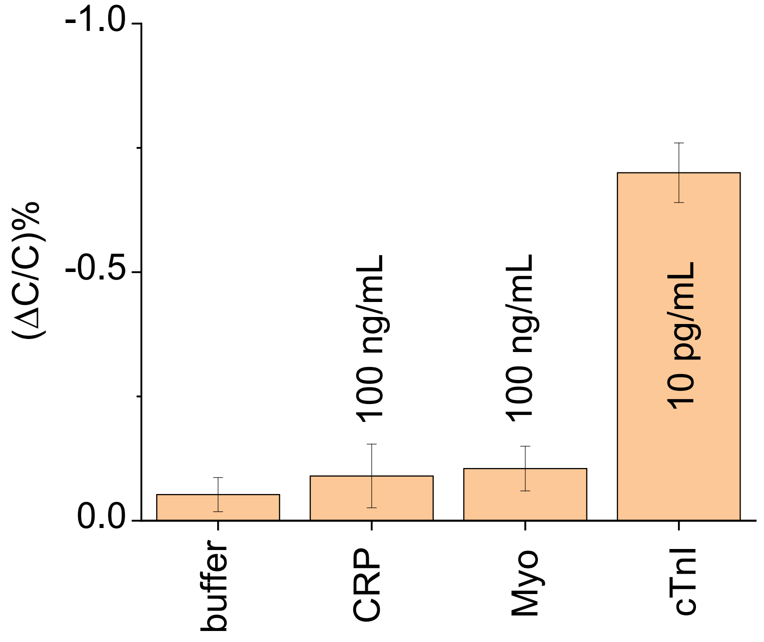

Figure S4. Selectivity analysis of the MiCaP biosensor in the presence of potential interfering biomarkers at elevated pathological concentrations. The sensor response was measured in the presence of 100 pg/mL cTnI (target analyte), 100 ng/mL C-reactive protein (CRP), and 100 ng/mL myoglobin in binding buffer. The capacitance changes in response to CRP and myoglobin remained within the baseline range, confirming minimal cross-reactivity and high specificity of the sensor under clinically relevant interference conditions.

- **Figure S5: Diagnostic potential of ISF for cTnI monitoring**

To validate the existence and estimation of cTnI concentration in ISF, we performed an experiment involving the extraction of dermal ISF using a suction blister technique and compared its concentration with serum cTnI. For this purpose, we first shaved the dorsal skin of a rat with a razor and hair removal cream, then thoroughly cleaned the skin with ethanol, running deionized water, and cleanroom wipes. A suction blister was applied to the region, and ISF extraction was allowed for 20–30 minutes. The extracted ISF was collected using a capillary tube, and the process was repeated multiple times to obtain the required amount of ISF. Blood samples were then collected from the tail vein of the rats, and serum was extracted through centrifugation for 10 minutes. Both ISF and serum samples were analyzed using the DXI 600 immunoassay system (Beckman Coulter, Switzerland), following standardized protocols to ensure accurate and reliable results. Figures S4a and b provide images of the dermal ISF extraction process, and the cTnI results in ISF and serum are provided in Figure S4c. The findings from our experiment and the supporting literature confirm that cTnI is present in interstitial fluid (ISF), with concentrations lower than those found in serum [82-87]. However, the experimental results indicate that the difference in cTnI concentrations between ISF and serum is not statistically significant. This underscores the reliability of ISF as a representative biofluid for cTnI monitoring. This further validates dermal ISF as a dependable and innovative medium for cTnI measurement.

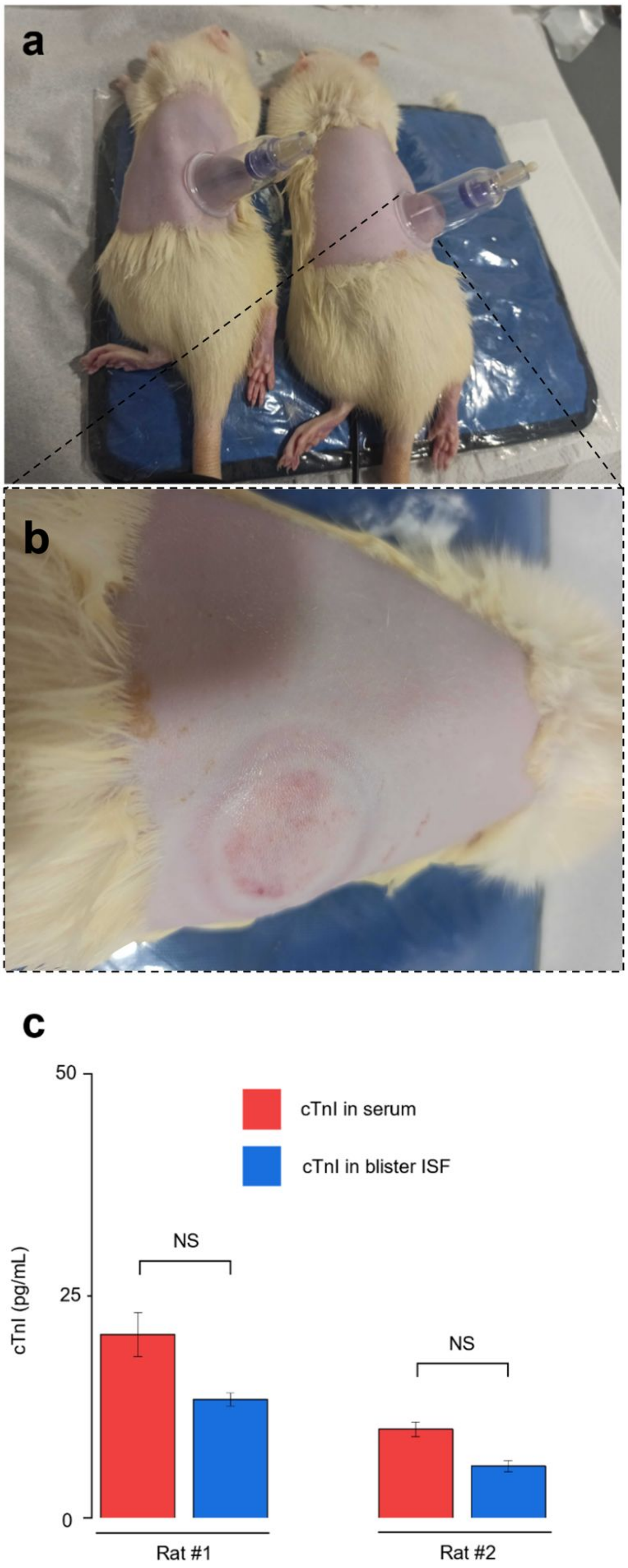

Figure S5. Experimental design for dermal ISF extraction and cTnI concentration comparison in serum and ISF. a) Suction blisters were attached to the two rats' shaved and

cleaned dorsal skin. b) Extracted ISF on the dorsal skin of a rat. The inset displays the dorsal skin after several applications of the suction blister for ISF extraction. c) Results of the cTnI concentration measurement in collected serum and blister ISF. The plot indicates no significant difference between the cTnI concentrations in serum and ISF, demonstrating that ISF can be reliably used as a biological solution for cTnI concentration measurement. Each experiment was repeated three times. Data were expressed as the mean ± SD. NS: no significant difference.

- **Supplementary Note 5:** Materials and Methods

Materials, reagents, and solutions

All chemicals and reagents used in this study were of analytical grade. Tris(hydroxymethyl)aminomethane (Tris), Tris-HCl, sodium chloride (NaCl), ethylenediaminetetraacetic acid disodium salt dihydrate (EDTA), urea, 6-Mercapto-1-hexanol (MCH), Tween® 40 (polyoxyethylene (20) sorbitan monopalmitate), cysteamine hydrochloride, and phosphate-buffered saline (PBS, sterile solution), and glutaraldehyde solution (50 wt.% in H_2O) was acquired from Sigma-Aldrich (USA). Bovine serum albumin (BSA) was purchased from ThermoFisher Scientific (Waltham, MA, USA). Monoclonal anti-cardiac troponin I antibody (mAb), human cardiac troponin I (cTnI, purity >95%), human NT-proBNP (purity >95%), human C-reactive protein (CRP, purity >95%), human cardiac troponin T (cTnT, purity >95%), myoglobin (purity >95%), human carcinoembryonic antigen (CEA), and cTnI-free human serum samples were obtained from HyTest Ltd. (Turku, Finland). Poly(lactic acid) (PLA) was sourced from Goodfellow (Cambridge, UK). The Sylgard® 184 silicone elastomer kit (10:1 ratio).

Instruments

The impedance characterization of the MiCaP was performed using an LCX meter (R&S®LCX200, Rohde and Schwarz) and an impedance analyzer (HIOKI, IM3536). CV and EIS was performed using potentiostat galvanostat equipment (Autolab, PGSTAT 101). The SEM images were taken by an ultra-plus field emission scanning electron microscope (Zeiss, EVO-LS15). Microneedle penetration depth experiment was performed by a confocal laser microscope (Olympus, LEXT OLS5000). The Cr/Au deposition was performed by thin film deposition system (Kurt J. Lesker PVD 75 PRO-Line). Plasma treatment of MiCaP was performed by a basic plasma cleaner (Harrick Plasma, PDC-32G). Optical images were taken

by stereo microscope (Leica S9i). Solution-depletion experiment for surface functionalization efficiency quantification was performed by a UV-VIS-NIR spectrophotometer (SHIMADZU, UV-3600i Plus). AFM images were acquired by Bruker Dimension Icon AFM.

Method for in vitro characterization

In this study, we conducted in vitro characterization of the MiCaP using an impedance analyzer operating at a frequency of 10 kHz and a signal amplitude of 30 mV. cTnI samples were prepared at concentrations of 10 pg/mL, 100 pg/mL, 1 ng/mL, and 10 ng/mL in 0.1× PBS containing 0.02% sodium azide. Anti-cTnI antibodies were prepared at 100 ng/mL in the same buffer and used for sensor functionalization. The functionalized MiCaP was placed in a custom-designed measurement chamber (Figure S2) and connected to the impedance analyzer. For each dose, 200 µL of the cTnI sample was introduced into the chamber, and capacitance was continuously measured for 15 minutes. For the selectivity experiments, each interfering protein was individually spiked into 0.1× PBS to a final concentration of 1 ng/mL. Each sample was then applied to a freshly functionalized MiCaP sensor, and capacitance was recorded using the same protocol as in the dose–response measurements.

In vivo experiments approval

The Koç University Research Center for Translational Medicine (KUTTAM) Committee approved all procedures for in vivo experiments (approval number: 2022.HADYEK.025). Wistar albino rats were housed in KUTTAM's Animal Research Facility and provided with food and water ad libitum while maintained under a 12-hour light-dark cycle, constant temperature (21-23°C), and humidity (45-50%).

Assessment of the blood composition and chemistry in rats

All experimental procedures in this study were conducted in accordance with the guidelines established by the Koç University Animal Care and Use Committee to ensure ethical and

treatment of the animals used in the study. Blood samples were obtained from Wistar albino rats (during the in vivo experiments), which had received saline serum/cTnI injection. Blood was collected via the tail vein immediately after the experiment. Blood was collected using gel tubes and centrifuged for serum extraction for serum chemistry testing. The serum measurements were performed by DXI 600 immunoassay system (Beckman Coulter, Switzerland).

Statistical analysis

The mean ± s.d. values were used to report the results. OriginLab software (v.9.65) was used to conduct statistical analyses. Each condition was tested in at least three biological replicates for all experiments. Statistical significance was assessed using t-tests. All data are expressed as mean ± SD (standard deviation). P-values of less than 0.05 ($p < 0.05$) indicated that the results were considered statistically significant. *$P < 0.05$, **$P < 0.005$, **$P < 0.001$.

References:

1. Fu, H.; Qin, Z.; Li, X.; Pan, Y.; Xu, H.; Pan, P.; Song, P.; Liu, X., Paper-Based All-in-One Origami Nanobiosensor for Point-of-Care Detection of Cardiac Protein Markers in Whole Blood. *ACS Sensors* **2023,** *8* (9), 3574-3584.
2. Chen, J. N.; Hasabnis, G. K.; Akin, E.; Gao, G.; Usha, S. P.; Süssmuth, R.; Altintas, Z., Developing innovative point-of-care electrochemical sensors empowered by cardiac troponin I-responsive nanocomposite materials. *Sensors and Actuators B: Chemical* **2024,** *417*, 136052.
3. Ma, J.; Feng, L.; Li, J.; Zhu, D.; Wang, L.; Su, S., Biological Recognition-Based Electrochemical Aptasensor for Point-of-Care Detection of cTnI. *Biosensors* **2023,** *13* (7), 746.
4. Choudhary, S.; Altintas, Z., Development of a Point-of-Care SPR Sensor for the Diagnosis of Acute Myocardial Infarction. *Biosensors* **2023,** *13* (2), 229.
5. Liu, J.; Ruan, G.; Ma, W.; Sun, Y.; Yu, H.; Xu, Z.; Yu, C.; Li, H.; Zhang, C.-w.; Li, L., Horseradish peroxidase-triggered direct in situ fluorescent immunoassay platform for sensing cardiac troponin I and SARS-CoV-2 nucleocapsid protein in serum. *Biosensors and Bioelectronics* **2022,** *198*, 113823.
6. Khachornsakkul, K.; Dungchai, W., Rapid Distance-Based Cardiac Troponin Quantification Using Paper Analytical Devices for the Screening and the Follow-Up of Acute Myocardial Infarction, Using a Single Drop of Human Whole Blood. *ACS Sensors* **2021,** *6* (3), 1339-1347.
7. Lee, T.-H.; Chen, L.-C.; Wang, E.; Wang, C.-C.; Lin, Y.-R.; Chen, W.-L., Development of an Electrochemical Immunosensor for Detection of Cardiac Troponin I at the Point-of-Care. *Biosensors* **2021,** *11* (7), 210.
8. Harpak, N.; Borberg, E.; Raz, A.; Patolsky, F., The “Bloodless” Blood Test: Intradermal Prick Nanoelectronics for the Blood Extraction-Free Multiplex Detection of Protein Biomarkers. *ACS Nano* **2022,** *16* (9), 13800-13813.
9. Li, P.-R.; Kiran Boilla, S.; Wang, C.-H.; Lin, P.-C.; Kuo, C.-N.; Tsai, T.-H.; Lee, G.-B., A self-driven, microfluidic, integrated-circuit biosensing chip for detecting four cardiovascular disease biomarkers. *Biosensors and Bioelectronics* **2024,** *249*, 115931.
10. Wang, L.; Xing, B.; Wang, H.; Hu, L.; Kuang, X.; Liang, H.; Wu, D.; Wei, Q., Electrochemiluminescence immunosensor based on the quenching effect of CuO@GO on m-CNNS for cTnI detection. *Analytical Biochemistry* **2021,** *612*, 114012.
11. Chen, H.; Li, Z.-y.; Chen, J.; Yu, H.; Zhou, W.; Shen, F.; Chen, Q.; Wu, L., CRISPR/Cas12a-based electrochemical biosensor for highly sensitive detection of cTnI. *Bioelectrochemistry* **2022,** *146*, 108167.
12. Cheng, D.; Zhou, Z.; Shang, S.; Wang, H.; Guan, H.; Yang, H.; Liu, Y., Electrochemical immunosensor for highly sensitive detection of cTnI via in-situ initiated ROP signal amplification strategy. *Analytica Chimica Acta* **2022,** *1219*, 340032.
13. Tannenberg, R.; Paul, M.; Röder, B.; Gande, S. L.; Sreeramulu, S.; Saxena, K.; Richter, C.; Schwalbe, H.; Swart, C.; Weller, M. G., Chemiluminescence Biosensor for the Determination of Cardiac Troponin I (cTnI). *Biosensors* **2023,** *13* (4), 455.
14. Yu, Z.; Lin, Q.; Gong, H.; Li, M.; Tang, D., Integrated solar-powered MEMS-based photoelectrochemical immunoassay for point-of-care testing of cTnI protein. *Biosensors and Bioelectronics* **2023,** *223*, 115028.
15. Khushaim, W.; Peramaiah, K.; Beduk, T.; Vijjapu, M. T.; Ilton de Oliveira Filho, J.; Huang, K.-W.; Mani, V.; Salama, K. N., Porous graphitic carbon nitrides integrated biosensor for sensitive detection of cardiac troponin I. *Biosensors and Bioelectronics: X* **2022,** *12*, 100234.
16. Saeidi, M.; Amidian, M. A.; Sheybanikashani, S.; Mahdavi, H.; Alimohammadi, H.; Syedmoradi, L.; Mohandes, F.; Zarrabi, A.; Tamjid, E.; Omidfar, K.; Simchi, A., Multilayered Mesoporous Composite Nanostructures for Highly Sensitive Label-Free Quantification of Cardiac Troponin-I. *Biosensors* **2022,** *12* (5), 337.

17. Chen, D.; Gong, Y.; Jin, Y., Detection of Cardiac Troponin I in Serum by CMK-3/AuNPs-based Electrochemical Sensor. *International Journal of Electrochemical Science* **2022,** *17* (7), 220716.
18. Kim, K. H.; Wee, K. W.; Kim, C.; Hur, D.; Lee, J. H.; Yoo, Y. K. Rapid and low-cost, and disposable electrical sensor using an extended gate field-effect transistor for cardiac troponin I detection *Biomed Eng Lett* [Online], 2022, p. 197-203. PubMed.

19. hui, H.; Gopinath, S. C. B.; Ismail, Z. H.; Chen, Y.; Pandian, K.; Velusamy, P., Cardiovascular biomarker troponin I biosensor: Aptamer-gold-antibody hybrid on a metal oxide surface. *Biotechnology and Applied Biochemistry* **2023,** *70* (2), 581-591.
20. Sun, B.; Bao, L.; Sun, Y.; Liu, J.; Wu, Y.; Li, H.; Yu, S.; Liu, Y.; Dang, Q.; Yang, L., Electrochemical immunosensor based on ferrocene derivatives amplified signal for detection of acute myocardial infarction warning biomarker-cTnI. *Microchemical Journal* **2024,** *199*, 110057.
21. Park, G.; Lee, H.; Jang, M.; Park, J. A.; Park, H.; Park, C.; Kim, T.-H.; Lee, M.-H.; Lee, T., Rapid electrical biosensor consisting of DNA aptamer/carbon nanonetwork on microelectrode array for cardiac troponin I in human serum. *Sensors and Actuators B: Chemical* **2023,** *393*, 134295.
22. Wang, S.; Tang, F.; Xing, S.; Xiang, S.; Dou, S.; Li, Y.; Liu, Q.; Wang, P.; Li, Y.; Feng, K.; Wang, S., An ultrasensitive electrochemical immunosensor based on meso-PdN NCs and Au NPs/N-CNTs for quantitative cTnI detection. *Bioelectrochemistry* **2024,** *158*, 108680.
23. Zhang, J.; Sun, K.; Ren, J.; Wang, H.; Cheng, J., An electrochemical metallic nanowire aptasensor for rapid and ultrasensitive detection of cardiac troponin I. *Sensors and Actuators B: Chemical* **2024,** *401*, 135001.
24. Nair, P.; Amreen, K.; Ponnalagu, R. N.; Goel, S., 3D Printed Interdigitated Electrodes for Cardiac Biomarker Detection. *IEEE Transactions on NanoBioscience* **2024**, 1-1.
25. Chen, X.; Zhang, C.; Liu, X.; Dong, Y.; Meng, H.; Qin, X.; Jiang, Z.; Wei, X., Low-noise fluorescent detection of cardiac troponin I in human serum based on surface acoustic wave separation. *Microsystems & Nanoengineering* **2023,** *9* (1), 141.
26. Memon, R.; Shaheen, I.; Qureshi, A.; Niazi, J. H., Enhanced detection of cardiac troponin-I using CdSe/CdS/ZnS core-shell quantum dot/TiO2 heterostructure photoelectrochemical sensor. *Journal of Alloys and Compounds* **2024,** *1008*, 176592.
27. Pan, T.-M.; Wang, C.-W.; Weng, W.-C.; Lai, C.-C.; Lu, Y.-Y.; Wang, C.-Y.; Hsieh, I. C.; Wen, M.-S., Rapid and label-free detection of the troponin in human serum by a TiN-based extended-gate field-effect transistor biosensor. *Biosensors and Bioelectronics* **2022,** *201*, 113977.
28. Khushaim, W.; Mani, V.; Peramaiya, K.; Huang, K.-W.; Salama, K. N., Ruthenium and Nickel Molybdate-Decorated 2D Porous Graphitic Carbon Nitrides for Highly Sensitive Cardiac Troponin Biosensor. *Biosensors* **2022,** *12* (10), 783.
29. Rauf, S.; Mani, V.; Lahcen, A. A.; Yuvaraja, S.; Beduk, T.; Salama, K. N., Binary transition metal oxide modified laser-scribed graphene electrochemical aptasensor for the accurate and sensitive screening of acute myocardial infarction. *Electrochimica Acta* **2021,** *386*, 138489.
30. Feng, S.; Yan, M.; Xue, Y.; Huang, J.; Yang, X., Electrochemical Immunosensor for Cardiac Troponin I Detection Based on Covalent Organic Framework and Enzyme-Catalyzed Signal Amplification. *Analytical Chemistry* **2021,** *93* (40), 13572-13579.
31. Gholami, M. D.; O'Mullane, A. P.; Sonar, P.; Ayoko, G. A.; Izake, E. L., Antibody coated conductive polymer for the electrochemical immunosensing of Human Cardiac Troponin I in blood plasma. *Analytica Chimica Acta* **2021,** *1185*, 339082.
32. Boonkaew, S.; Jang, I.; Noviana, E.; Siangproh, W.; Chailapakul, O.; Henry, C. S., Electrochemical paper-based analytical device for multiplexed, point-of-care detection of cardiovascular disease biomarkers. *Sensors and Actuators B: Chemical* **2021,** *330*, 129336.

33. Gupta, A.; Sharma, S. K.; Pachauri, V.; Ingebrandt, S.; Singh, S.; Sharma, A. L.; Deep, A., Sensitive impedimetric detection of troponin I with metal–organic framework composite electrode. *RSC Advances* **2021,** *11* (4), 2167-2174.
34. Gupta, A.; Kumar Sharma, S.; L. Sharma, A.; Deep, A., 2-Aminotrimesic Acid-Functionalized Graphene Oxide-Modified Screen-Printed Electrodes for Sensitive Electrochemical Detection of Cardiac Marker Troponin I. *physica status solidi (a)* **2021,** *218* (13), 2000700.
35. Li, J.; Zhang, S.; Zhang, L.; Zhang, Y.; Zhang, H.; Zhang, C.; Xuan, X.; Wang, M.; Zhang, J.; Yuan, Y., A Novel Graphene-Based Nanomaterial Modified Electrochemical Sensor for the Detection of Cardiac Troponin I. *Frontiers in Chemistry* **2021,** *9*.
36. Wang, Y.; Singh, R.; Li, M.; Min, R.; Wu, Q.; Kaushik, B. K.; Jha, R.; Zhang, B.; Kumar, S., Cardiac Troponin I Detection Using Gold/Cerium-Oxide Nanoparticles Assisted Hetro-Core Fiber Structure. *IEEE Transactions on NanoBioscience* **2023,** *22* (2), 375-382.
37. Toma, K.; Oishi, K.; Iitani, K.; Arakawa, T.; Mitsubayashi, K., Surface plasmon-enhanced fluorescence immunosensor for monitoring cardiac troponin I. *Sensors and Actuators B: Chemical* **2022,** *368*, 132132.
38. Kitte, S. A.; Bushira, F. A.; Soreta, T. R., An impedimetric aptamer-based sensor for sensitive and selective determination of cardiac troponin I. *Journal of the Iranian Chemical Society* **2022,** *19* (2), 505-511.
39. Wang, L.; Han, Y.; Wang, H.; Han, Y.; Liu, J.; Lu, G.; Yu, H., A MXene-functionalized paper-based electrochemical immunosensor for label-free detection of cardiac troponin I. *Journal of Semiconductors* **2021,** *42* (9), 092601.
40. Wang, S.; Qin, J.; Liang, Y.; Ye, Y.; Li, S.; Guo, Y.; Yang, X.; Liang, Y., A sensitive Raman spectroscopy sensor for determination cardiac troponin I based on proteolytic peptide magnetic imprinting technology. *Microchemical Journal* **2024,** *196*, 109610.
41. Wang, H.; Lu, Q.; Luo, J.; Zeng, X.; Zhao, C.; Du, F.; Zhang, Y.; Zeng, G.; Zhang, S., Photoelectrochemical determination of cardiac troponin I based on rod-like g-C3N5@MnO2 heterostructure. *Microchimica Acta* **2022,** *190* (1), 19.
42. Shkhair, A. I.; Madanan, A. S.; Varghese, S.; Abraham, M. K.; Indongo, G.; Rajeevan, G.; Arathy, B. K.; Abbas, S. M.; George, S., Nickel Nanocluster as a Fluorescent Probe for the Non-enzymatic Detection of Cardiac Troponin I. *Plasmonics* **2024**.
43. Cen, S.-Y.; Ge, X.-Y.; Chen, Y.; Wang, A.-J.; Feng, J.-J., Label-free electrochemical immunosensor for ultrasensitive determination of cardiac troponin I based on porous fluffy-like AuPtPd trimetallic alloyed nanodendrites. *Microchemical Journal* **2021,** *169*, 106568.
44. Wang, T.; Tan, H.-S.; Zhao, L.-X.; Liu, M.; Li, S.-S., A novel ratiometric aptasensor based on SERS for accurate quantification of cardiac troponin I. *Sensors and Actuators B: Chemical* **2024,** *412*, 135804.
45. Meng, Y.; Li, Y.; Liu, S.; Wang, S.; Dong, H.; Jiang, F.; Liu, Q.; Li, Y.; Wei, Q., Sandwich-type electrochemical immunosensor based on CuFe2O4-Pd for cardiac troponin I detection. *Microchimica Acta* **2023,** *190* (6), 249.
46. Zeng, L.; Lin, C.; Liu, P.; Sun, D.; Lu, J., Anisotropic aptamer-modified DNA tetrahedra/MOF nanoprobes for enhanced colorimetric aptasensing of cardiac troponin I. *Chemical Engineering Journal* **2023,** *474*, 145525.
47. Han, Y.; Su, X.; Fan, L.; Liu, Z.; Guo, Y., Electrochemical aptasensor for sensitive detection of Cardiac troponin I based on CuNWs/MoS2/rGO nanocomposite. *Microchemical Journal* **2021,** *169*, 106598.
48. Poursharifi, N.; Hassanpouramiri, M.; Zink, A.; Ucuncu, M.; Parlak, O., Transdermal Sensing of Enzyme Biomarker Enabled by Chemo-Responsive Probe-Modified Epidermal Microneedle Patch in Human Skin Tissue. *Advanced Materials* **2024,** *36* (30), 2403758.
49. Dervisevic, M.; Harberts, J.; Sánchez-Salcedo, R.; Voelcker, N. H., 3D Polymeric Lattice Microstructure-Based Microneedle Array for Transdermal Electrochemical Biosensing. *Advanced Materials* **2024,** *36* (48), 2412999.
50. Bakhshandeh, F.; Zheng, H.; Barra, N. G.; Sadeghzadeh, S.; Ausri, I.; Sen, P.; Keyvani, F.; Rahman, F.; Quadrilatero, J.; Liu, J., Wearable aptalyzer integrates microneedle

and electrochemical sensing for in vivo monitoring of glucose and lactate in live animals. *Advanced Materials* **2024,** *36* (35), 2313743.
51. Ausri, I. R.; Sadeghzadeh, S.; Biswas, S.; Zheng, H.; GhavamiNejad, P.; Huynh, M. D. T.; Keyvani, F.; Shirzadi, E.; Rahman, F. A.; Quadrilatero, J., Multifunctional Dopamine-Based Hydrogel Microneedle Electrode for Continuous Ketone Sensing. *Advanced Materials* **2024,** *36* (32), 2402009.
52. Huang, X.; Zheng, S.; Liang, B.; He, M.; Wu, F.; Yang, J.; Chen, H.-j.; Xie, X., 3D-assembled microneedle ion sensor-based wearable system for the transdermal monitoring of physiological ion fluctuations. *Microsystems & Nanoengineering* **2023,** *9* (1), 25.
53. Lin, S.; Cheng, X.; Zhu, J.; Wang, B.; Jelinek, D.; Zhao, Y.; Wu, T.-Y.; Horrillo, A.; Tan, J.; Yeung, J., Wearable microneedle-based electrochemical aptamer biosensing for precision dosing of drugs with narrow therapeutic windows. *Science advances* **2022,** *8* (38), eabq4539.
54. Molinero-Fernández, Á.; Casanova, A.; Wang, Q.; Cuartero, M.; Crespo, G. A., In vivo transdermal multi-ion monitoring with a potentiometric microneedle-based sensor patch. *ACS sensors* **2022,** *8* (1), 158-166.
55. Tehrani, F.; Teymourian, H.; Wuerstle, B.; Kavner, J.; Patel, R.; Furmidge, A.; Aghavali, R.; Hosseini-Toudeshki, H.; Brown, C.; Zhang, F., An integrated wearable microneedle array for the continuous monitoring of multiple biomarkers in interstitial fluid. *Nature Biomedical Engineering* **2022,** *6* (11), 1214-1224.
56. Wang, Q.; Molinero-Fernandez, A.; Casanova, A.; Titulaer, J.; Campillo-Brocal, J. C.; Konradsson-Geuken, Å.; Crespo, G. A.; Cuartero, M., Intradermal glycine detection with a wearable microneedle biosensor: the first in vivo assay. *Analytical Chemistry* **2022,** *94* (34), 11856-11864.
57. Yang, B.; Wang, H.; Kong, J.; Fang, X., Long-term monitoring of ultratrace nucleic acids using tetrahedral nanostructure-based NgAgo on wearable microneedles. *Nature Communications* **2024,** *15* (1), 1936.
58. Yang, B.; Kong, J.; Fang, X., Programmable CRISPR-Cas9 microneedle patch for long-term capture and real-time monitoring of universal cell-free DNA. *Nature Communications* **2022,** *13* (1), 3999.
59. Keum, D. H.; Jung, H. S.; Wang, T.; Shin, M. H.; Kim, Y.-E.; Kim, K. H.; Ahn, G. O.; Hahn, S. K., Microneedle biosensor for real-time electrical detection of nitric oxide for in situ cancer diagnosis during endomicroscopy. *Advanced healthcare materials* **2015,** *4* (8), 1153-1158.
60. Moonla, C.; Reynoso, M.; Casanova, A.; Chang, A.-Y.; Djassemi, O.; Balaje, A.; Abbas, A.; Li, Z.; Mahato, K.; Wang, J., Continuous ketone monitoring via wearable microneedle patch platform. *ACS sensors* **2024,** *9* (2), 1004-1013.
61. Goud, K. Y.; Moonla, C.; Mishra, R. K.; Yu, C.; Narayan, R.; Litvan, I.; Wang, J., Wearable electrochemical microneedle sensor for continuous monitoring of levodopa: toward Parkinson management. *ACS sensors* **2019,** *4* (8), 2196-2204.
62. Dervisevic, M.; Jara Fornerod, M. J.; Harberts, J.; Zangabad, P. S.; Voelcker, N. H., Wearable microneedle patch for transdermal electrochemical monitoring of urea in interstitial fluid. *ACS sensors* **2024,** *9* (2), 932-941.
63. Freeman, D. M.; Ming, D. K.; Wilson, R.; Herzog, P. L.; Schulz, C.; Felice, A. K.; Chen, Y.-C.; O'Hare, D.; Holmes, A. H.; Cass, A. E., Continuous measurement of lactate concentration in human subjects through direct electron transfer from enzymes to microneedle electrodes. *ACS sensors* **2023,** *8* (4), 1639-1647.
64. Singh, N.; Zhang, Q.; Xu, W.; Whitham, S. A.; Dong, L., A Biohydrogel-Enabled Microneedle Sensor for In Situ Monitoring of Reactive Oxygen Species in Plants. *ACS sensors* **2025,** *10* (3), 1797-1810.
65. Yang, J.; Gong, X.; Chen, S.; Zheng, Y.; Peng, L.; Liu, B.; Chen, Z.; Xie, X.; Yi, C.; Jiang, L., Development of smartphone-controlled and microneedle-based wearable continuous glucose monitoring system for home-care diabetes management. *ACS sensors* **2023,** *8* (3), 1241-1251.

66. Luo, X.; Yu, Q.; Yang, L.; Cui, Y., Wearable, sensing-controlled, ultrasound-based microneedle smart system for diabetes management. *ACS sensors* **2023,** *8* (4), 1710-1722.
67. Liu, Z.; Huang, X.; Liu, Z.; Zheng, S.; Yao, C.; Zhang, T.; Huang, S.; Zhang, J.; Wang, J.; Farah, S., Plug-In Design of the Microneedle Electrode Array for Multi-Parameter Biochemical Sensing in Gouty Arthritis. *ACS sensors* **2025**.
68. Wang, Q.; Molinero-Fernandez, Á.; Wei, Q.; Xuan, X.; Konradsson-Geuken, Å.; Cuartero, M.; Crespo, G. A., Intradermal Lactate Monitoring Based on a Microneedle Sensor Patch for Enhanced In Vivo Accuracy. *ACS sensors* **2024,** *9* (6), 3115-3125.
69. Molinero-Fernandez, Á.; Wang, Q.; Xuan, X.; Konradsson-Geuken, Å.; Crespo, G. A.; Cuartero, M., Demonstrating the Analytical Potential of a Wearable Microneedle-Based Device for Intradermal CO2 Detection. *ACS sensors* **2024,** *9* (1), 361-370.
70. Favaro, M.; Jeong, B.; Ross, P. N.; Yano, J.; Hussain, Z.; Liu, Z.; Crumlin, E. J., Unravelling the electrochemical double layer by direct probing of the solid/liquid interface. *Nature communications* **2016,** *7* (1), 12695.
71. Park, S.; McDaniel, J. G., Generalized Helmholtz model describes capacitance profiles of ionic liquids and concentrated aqueous electrolytes. *The Journal of Chemical Physics* **2024,** 160(16).
72. Kant, R.; Singh, M. B., Generalization of the Gouy-Chapman-Stern model of an electric double layer for a morphologically complex electrode: Deterministic and stochastic morphologies. *Physical Review E* **2013,** *88* (5).
73. Brown, M. A.; Bossa, G. V.; May, S., Emergence of a Stern Layer from the Incorporation of Hydration Interactions into the Gouy–Chapman Model of the Electrical Double Layer. *Langmuir* **2015,** *31* (42).
74. Yorganci, E.; Akyilmaz, E., Alkaline phosphatase based amperometric biosensor immobilized by cysteamine-glutaraldehyde modified self-assembled monolayer. *Artificial Cells, Blood Substitutes, and Biotechnology* **2011,** *39* (5), 317-323.
75. Sahajpal, K.; Shekhar, S.; Kumar, A.; Sharma, B.; Meena, M. K.; Bhagi, A. K.; Sharma, S., Dynamic protein and polypeptide hydrogels based on Schiff base co-assembly for biomedicine. *Journal of Materials Chemistry B* **2022,** *10* (17), 3173-3198.
76. Mera, K.; Nagai, M.; Brock, J. W.; Fujiwara, Y.; Murata, T.; Maruyama, T.; Baynes, J. W.; Otagiri, M.; Nagai, R., Glutaraldehyde is an effective cross-linker for production of antibodies against advanced glycation end-products. *Journal of immunological methods* **2008,** *334* (1-2), 82-90.
77. Ionescu, R. E., Use of cysteamine and glutaraldehyde chemicals for robust functionalization of substrates with protein biomarkers—an overview on the construction of biosensors with different transductions. *Biosensors* **2022,** *12* (8), 581.
78. Li, L.; Wang, S.; Xiao, Y.; Wang, Y., Recent advances in immobilization strategies for biomolecules in sensors using organic field-effect transistors. *Transactions of Tianjin University* **2020,** *26*, 424-440.
79. Abdulkarim, H.; Siaj, M., Label-free multiplex electrochemical immunosensor for early diagnosis of lysosomal storage disorders. *Scientific reports* **2022,** *12* (1), 9334.
80. Mirzajani, H.; Cheng, C.; Vafaie, R. H.; Wu, J.; Chen, J.; Eda, S.; Aghdam, E. N.; Ghavifekr, H. B., Optimization of ACEK-enhanced, PCB-based biosensor for highly sensitive and rapid detection of bisphenol a in low resource settings. *Biosensors and Bioelectronics* **2022,** *196*, 113745.
81. Aleman, J.; Kilic, T.; Mille, L. S.; Shin, S. R.; Zhang, Y. S., Microfluidic integration of regeneratable electrochemical affinity-based biosensors for continual monitoring of organ-on-a-chip devices. *Nature protocols* **2021,** *16* (5), 2564-2593.
82. Vermeer, B. J.; Reman, F. C.; van Gent, C. M., The determination of lipids and proteins in suction blister fluid. *Journal of Investigative Dermatology* **1979,** *73* (4).
83. Svedman, C.; Yu, B. B.; Ryan, T. J.; Svensson, H., Plasma proteins in a standardised skin mini-erosion (I): permeability changes as a function of time. *BMC dermatology* **2002,** *2*, 1-7.
84. Miller, P. R.; Taylor, R. M.; Tran, B. Q.; Boyd, G.; Glaros, T.; Chavez, V. H.; Krishnakumar, R.; Sinha, A.; Poorey, K.; Williams, K. P., Extraction and biomolecular analysis

of dermal interstitial fluid collected with hollow microneedles. *Communications biology* **2018,** *1* (1), 173.
85. Samant, P. P.; Prausnitz, M. R., Mechanisms of sampling interstitial fluid from skin using a microneedle patch. *Proceedings of the National Academy of Sciences* **2018,** *115* (18), 4583-4588.
86. Schultze, A. E.; Carpenter, K. H.; Wians, F. H.; Agee, S. J.; Minyard, J.; Lu, Q. A.; Todd, J.; Konrad, R. J., Longitudinal studies of cardiac troponin-I concentrations in serum from male Sprague Dawley rats: baseline reference ranges and effects of handling and placebo dosing on biological variability. *Toxicologic pathology* **2009,** *37* (6), 754-760.
87. Heikenfeld, J.; Jajack, A.; Feldman, B.; Granger, S. W.; Gaitonde, S.; Begtrup, G.; Katchman, B. A., Accessing analytes in biofluids for peripheral biochemical monitoring. *Nature biotechnology* **2019,** *37* (4), 407-419.
